# Evi5 is required for *Xenopus* limb and tail regeneration

**DOI:** 10.3389/fcell.2022.1027666

**Published:** 2022-12-08

**Authors:** Li Yang, Youwei Chen, Huahua Liu, Yu Liu, Feng Yuan, Qianyan Li, Gufa Lin

**Affiliations:** Key Laboratory of Spine and Spinal Cord Injury Repair and Regeneration of Ministry of Education, Orthopaedic Department of Tongji Hospital, Research Center for Translational Medicine Shanghai East Hospital, School of Life Sciences and Technology, Tongji University, Shanghai, China

**Keywords:** Evi5, regeneration, limb, tail, *Xenopus*, axolotl, Kdm6b, Kdm7a

## Abstract

Amphibians such as salamanders and the African clawed frog *Xenopus* are great models for regeneration studies because they can fully regenerate their lost organs. While axolotl can regenerate damaged organs throughout its lifetime, *Xenopus* has a limited regeneration capacity after metamorphosis. The ecotropic viral integrative factor 5 (Evi5) is of great interest because its expression is highly upregulated in the limb blastema of axolotls, but remains unchanged in the fibroblastema of post-metamorphic frogs. Yet, its role in regeneration-competent contexts in *Xenopus* has not been fully analyzed. Here we show that Evi5 is upregulated in *Xenopus* tadpoles after limb and tail amputation, as in axolotls. Down-regulation of Evi5 with morpholino antisense oligos (Mo) impairs limb development and limb blastema formation in *Xenopus* tadpoles. Mechanistically, we show that Evi5 knockdown significantly reduces proliferation of limb blastema cells and causes apoptosis, blocking the formation of regeneration blastema. RNA-sequencing analysis reveals that in addition to reduced PDGFα and TGFβ signaling pathways that are required for regeneration, *evi5* Mo downregulates lysine demethylases Kdm6b and Kdm7a. And knockdown of Kdm6b or Kdm7a causes defective limb regeneration. Evi5 knockdown also impedes tail regeneration in *Xenopus* tadpoles and axolotl larvae, suggesting a conserved function of Evi5 in appendage regeneration. Thus, our results demonstrate that Evi5 plays a critical role in appendage regeneration in amphibians.

## Introduction

Many amphibian animals have the ability to regenerate all or portions of their appendages, including the limb and the tail (Carlson, 2007; Simon and Tanaka, 2013). Understanding the cellular and molecular mechanisms of amphibian appendage regeneration may eventually instruct mammalian limb regeneration (Cox et al., 2019; Davidian and Levin, 2022). The urodele amphibians axolotl (*Ambystoma mexicanum*) and newts (*Notophthalmus viridescens*) are primary salamander models of limb regeneration for querying the cellular and molecular signals that lead to a successful regeneration (Bassat and Tanaka, 2021). They can regenerate the limb from any level of amputation by forming a proliferative mass called blastema, which subsequently proliferates and differentiates to restore the lost structure. Limbs of anuran amphibians, such as *Xenopus,* can also regenerate. When the limb buds are amputated in young *Xenopus* tadpoles, such as those of Nieuwkoop-Faber (NF) stages 51–53 (Nieuwkoop and Faber, 1967), they regenerate perfectly well (Dent, 1962). However, the regenerative capacity of the tadpole limb becomes progressively decreased and restricted to more distal levels, after NF stage 53. At NF stage 56 or 57, amputation at any level results only in the regeneration of a muscle-less, un-segmented cartilage spike covered by an envelope of skin (Dent, 1962). Spike formation is also the default outcome of limb amputations in post-metamorphic *Xenopus* (Satoh et al., 2006). Following amputation of the *Xenopus* froglet limb, a fibrotic blastema (fibroblastema) is formed underneath the wound epithelium. Unlike salamander limb blastema cells, which undergo substantial dedifferentiation to embryonic limb bud states, the fibroblastema cells in *Xenopus* are only partially dedifferentiated (Gerber et al., 2018; Lin et al., 2021). Nevertheless, the age-dependent limb regeneration phenomenon makes *Xenopus* an interesting model of appendage regeneration (Slack et al., 2008). Comparative analysis of *Xenopus* and axolotl limb regeneration has also been useful for identifying factors mediating successful regeneration (Stocum and Cameron, 2011).

One such comparative study, from the Stocum group, profiled the proteomics of axolotl and *Xenopus* froglet limb blastema cells (Rao et al., 2014). This study identified Evi5 (ecotropic viral integrative factor 5) as one molecule of special interest in limb regeneration. EVI5 was strongly upregulated, with more than two-fold changes, at all stages during blastema formation in the axolotl (Rao et al., 2009). But its expression remained unchanged in the *Xenopus* limb fibroblastema (Rao et al., 2014). Evi5 is an oncoprotein involved in cell cycle regulation, and interacts with many cell cycle proteins (Lim and Tang, 2013). For example, Evi5 binds to and stabilizes the mitotic regulator Emi1 to prevent cells from entering mitosis prematurely (Eldridge et al., 2006). Emi1, the early mitotic inhibitor initially identified from *Xenopus* oocytes, accumulates in late G1 and inhibits cyclin A degradation by the anaphase-promoting complex/cyclosome (APC/C) (Reimann et al., 2001; Eldridge et al., 2006). The high level of expression of EVI5 protein in the regenerative axolotl limb blastema led to the postulation that it may have an important role in appendage regeneration (Rao et al., 2014). However, it is not clear whether Evi5 itself is functionally required for limb regeneration, and whether it is differentially expressed in regeneration-competent young *Xenopus* limbs. Neither has the mechanism of Evi5 in amphibian limb regeneration been investigated.

In this study, we have investigated the expression dynamics of *evi5* during limb and tail regeneration in young *Xenopus* tadpoles. Loss-of-function analyses revealed that Evi5 plays a critical role during appendage regeneration, inhibiting cell proliferation and causing apoptosis in the blastema cells. RNA sequencing analysis identified the potential downstream targets of Evi5 during limb regeneration. Aside from interfering with the cell cycle, knockdown of Evi5 inhibits the expression of lysine demethylases *kdm6b* and *kdm7a* in the regenerating tadpole limb, providing important clues for our further understanding of the mechanism of amphibian appendage regeneration.

## Results

### Expression of *evi5* in *Xenopus* tadpole limb and tail regeneration

**FIGURE 1:**
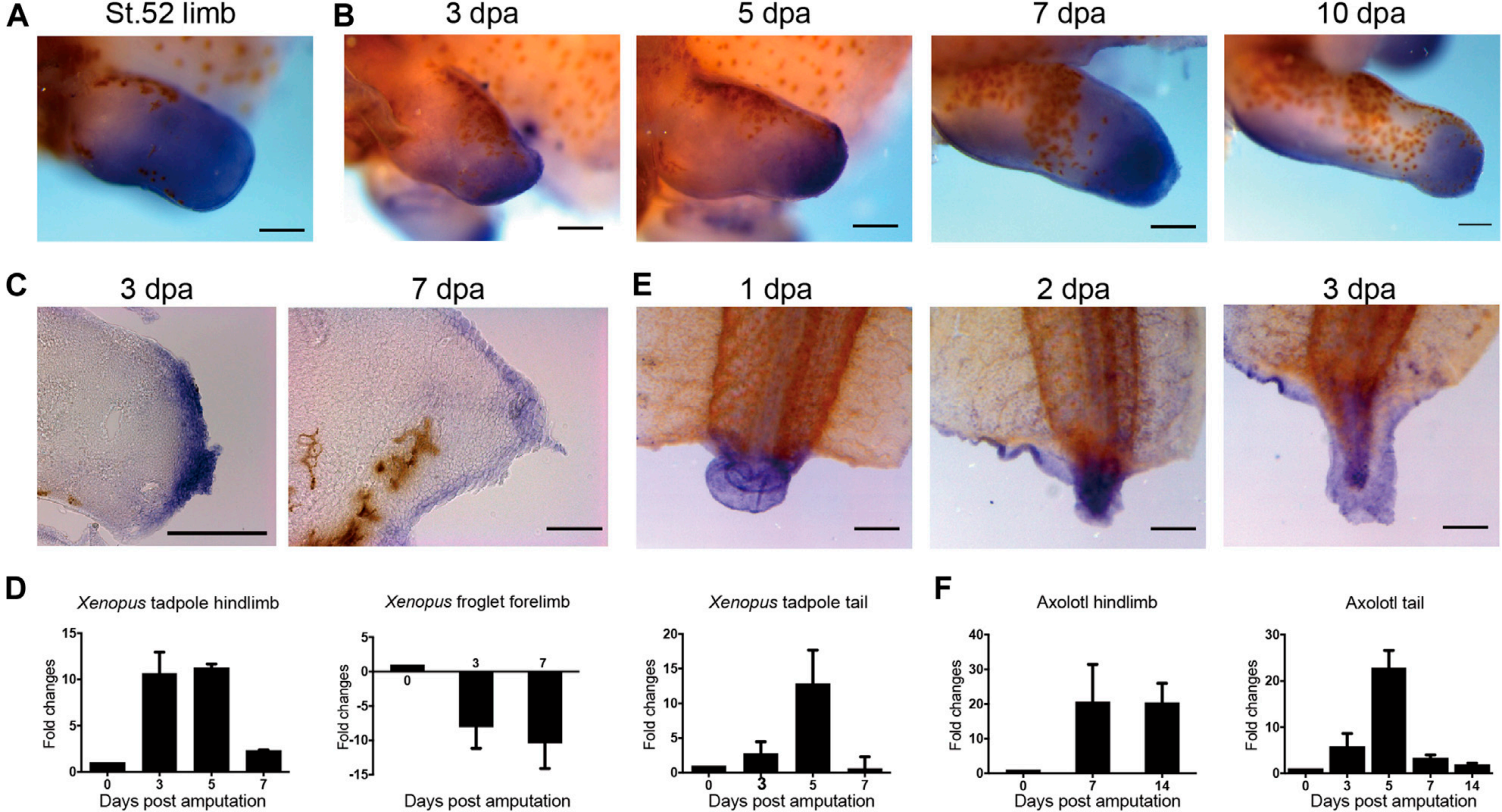
Expression of *evi5* during limb and tail regeneration in *Xenopus* and axolotl. **(A)** Detection of *evi5* mRNA in NF stage 52 tadpole hindlimb by whole mount *in situ* hybridization (WISH). **(B)** Detection of *evi5* mRNA in stage 52 tadpole hindlimb at 3,5,7 and 10 days post amputation (dpa). **(C)** Sagittal section of 3 and 7 dpa WISH specimens showing *evi5* expression in wound epithelium and blastema region of regenerating *Xenopus* tadpole hindlimb. **(D)** RT-PCR analysis of *evi5* in the NF stage 52 tadpole limb, froglet forelimb and tadpole tail. **(E)** WISH analysis of *evi5* mRNA expression during *Xenopus* tadpole tail regeneration. **(F)** RT-PCR analysis of *Evi5* in the regenerating axolotl limb and tail. Scale bars represent 500 μm in **(A–B,E)** and 3 dpa specimen in **(C)**, 50 μm in 7 dpa specimen shown in **(C)**. Data shown in **(D,F)** are mean with standard derivations, from 3 independent experiments, all with significant differences (*p* <0.05) analyzed with one-way ANOVA test.

The significant upregulation of EVI5 protein levels in axolotl limb blastema but not in the non-regenerating *Xenopus* froglet fibroblastema suggested a strong correlation between EVI5 expression dynamics and appendage regeneration ability (Rao et al., 2014). To further investigate the role of Evi5 in *Xenopus* appendage regeneration, we obtained the coding sequences of *Xenopus evi5* based on information found on Xenbase.org (see method). Protein sequence alignment showed that *Xenopus* and axolotl Evi5 proteins are highly conserved to both human and mouse EVI5 (Supplementary Figure S1). By whole-mount *in situ* hybridization (WISH), we examined the expression of *evi5* transcripts during *Xenopus* limb bud development. *evi5* was highly expressed in NF stage 52 limb bud (Figure 1A). This expression pattern is consistent with the observed high level of *Evi5* mRNA in the developing mouse limb (E10.5, http://www.informatics.jax.org/image/MGI:3501258) (Gray et al., 2004), suggesting that Evi5 has a role in the development of *Xenopus* limbs.

To examine the expression of *evi5* during *Xenopus* tadpole limb regeneration, we performed hindlimb amputation of NF stage 52–53 tadpoles, and detected *evi5* expression by WISH and RT-PCR at 3, 5, 7, and 10 days post-amputation (dpa). The results showed that *evi5* is highly upregulated during tadpole limb regeneration (Figure 1B). Paraffin sections collected from WISH specimens showed strong expression of *evi5* mRNA in the wound epithelium and the blastemal mesenchyme in the regenerating limb (Figure 1C). RT-PCR results confirmed that *evi5* mRNA level was most elevated in 3-5 dpa samples, corresponding to a time frame of blastema formation (Figure 1D). However, *evi5* mRNA levels did not increase, but decreased after amputation of the regeneration-deficient froglet forelimb (Figure 1D), in agreement with reported lack of expression of Evi5 protein in the fibroblastema of *Xenopus* froglet (Rao et al., 2014).

### Knockdown of Evi5 impairs *Xenopus* tadpole limb development and inhibits limb blastema formation

**FIGURE 2:**
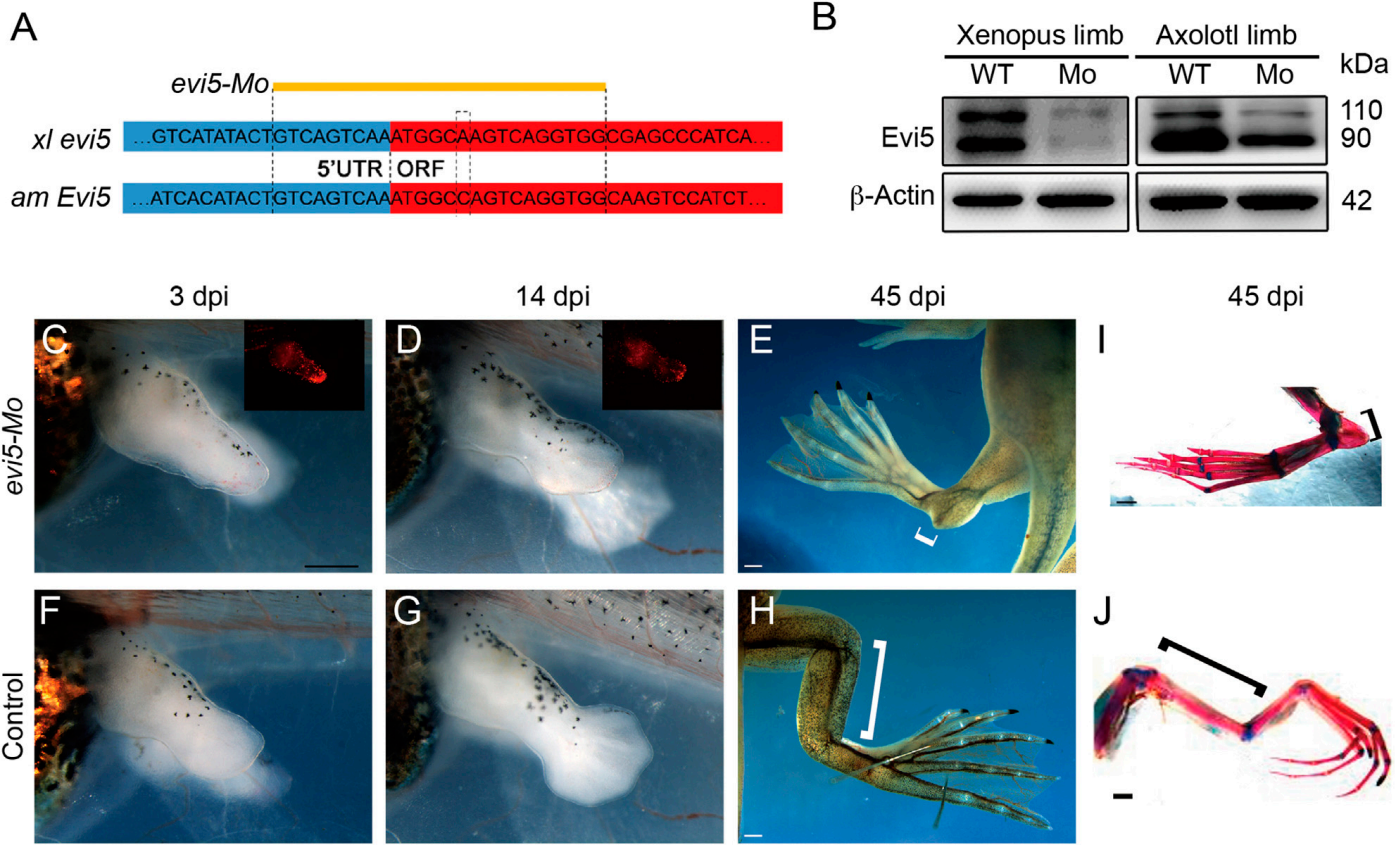
Effect of *evi5* Mo on limb development of *Xenopus*. **(A)** Design of *evi5* Mo against *Xenopus evi5* (*xl evi5*) and axolotl *Evi5* (*am Evi5*). Open reading frame indicated in red, targeted region of *evi5* Mo shown in yellow. **(B)** Detection of Evi5 protein by Western blotting, in *Xenopus* and axolotl limb stump after injection and electroporation of *evi5* Mo. Anti-Evi5 (ab 70790) was used for Evi5 protein. β-Actin used as loading control. **(C–H)** Knockdown of Evi5 expression during limb development. The Mo-injected limbs **(C–E)** showed significant developmental delay compared with the control group **(F–H)**, and developmental abnormalities were observed at late developmental stages (white brackets indicate the length of the zeugopods). **(I,J)** Skeletal staining of hindlimbs at late developmental stages, with red indicating bone tissue and blue indicating cartilage tissue. Black brackets indicate zeugopods. Scale bars represent 500 μm in **(C,D,F,G)** and 1 mm in **(E,H)** and **(I,J)**.

We designed a translation-blocking morpholino oligo (Mo) that recognizes both *Xenopus laevis evi5.L* and *evi5.S* for Evi5 protein knockdown experiments (Figure 2A). The Mo was modified with 3’-lissamine as a red-emitting fluorescent tag for easy visualization and tracing. We verified the efficacy of this Mo through electroporation into NF stage 52/53 tadpole limbs followed by Western blotting analysis, with an anti-Evi5 antibody (abcam 70790). The Western blotting result showed that *evi5* Mo could successfully decrease Evi5 protein levels in *Xenopus* tadpole limbs*.* The *evi5* Mo also targeted axolotl *Evi5* mRNA with one base mismatch, and could moderately decrease EVI5 protein in the axolotl limb (Figure 2B), probably due to less efficient delivery of Mo into the axolotl limb once all the digits have formed. We thus concluded that the *evi5* Mo could be used for Evi5 knockdown in *Xenopus* tadpole limbs.

We tested the specificity of the *evi5*-Mo in *Xenopus* embryos by co-injection of *evi5* mRNA and Mo. Injection of 20 ng of *evi5* Mo into one of the animal blastomeres of 4-8 cell stage embryos caused eye defects and a severely bent body axis at the late tailbud stage. The eye on the injected side was smaller and even absent (Supplementary Figures S2A–D,F,G), and the body axis was bending toward the injected side (Supplementary Figure S2E). However, co-injection of *evi5* mRNA together with *evi5* Mo could rescue the developmental abnormalities, thus demonstrating the specificity of *evi5* Mo (Supplementary Figures S2H,I).

We then injected and electroporated *evi5* Mo into one side of the hindlimb of stage 52–53 tadpoles, with control Mo or *GFP* DNA plasmid injected into the other side as controls (Supplementary Figure S3). We first followed the tadpole limb development, as *evi5* is also highly expressed in the developing *Xenopus* and mouse limb (Figure 1A and http://www.informatics.jax.org/image/MGI:3501258). We found significant retardation in the development of *evi5* Mo-injected tadpole limbs. At 14 days post injection (dpi), while the control limb had developed beyond NF stage 54, the Mo-injected limb still resembled an NF 52/53 limb (Figures 2C,D versus Figures 2F,G). At 45 dpi, the injected limbs of the post-metamorphic froglets were smaller and malformed, with shortened zeugopod segment (Figures 2E,I). Skeleton preparation revealed that the injected limb had a malformed tibia/fibula, although the overall patterning of the autopod was not affected (Figures 2I,J).

**FIGURE 3:**
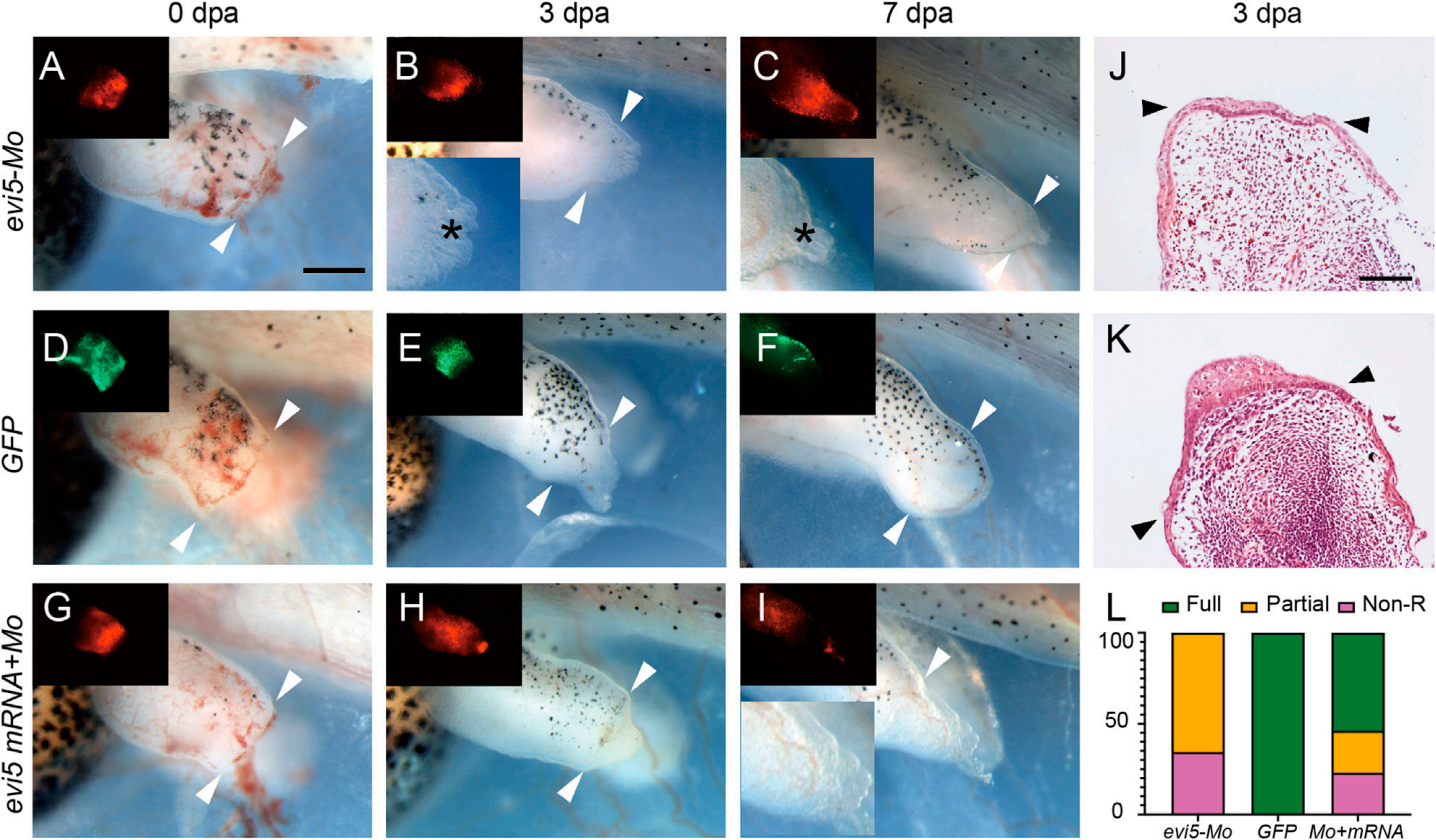
*Xenopus* tadpole limb regeneration after Evi5 knockdown. **(A–I)** Stage 52–53 tadpole hindlimbs at 0, 3 and 7 dpa, after injection/electroporation of *evi5* Mo **(A–C)**, *GFP* DNA **(D–F)**, or *evi5* mRNA + *evi5* Mo **(G–I)**. Fluorescent images (insets) indicate site of injected materials. Bright field insets show enlarged views of the limb stump. Black * indicates the epithelium layer formed in *evi5* Mo-injected tadpole limbs. White arrowheads indicate amputation levels. Scale bar: 0.5 mm. **(J**,**K)** HE staining of the *evi5* Mo and GFP-injected 3 dpa limb regenerates, showing that blastema formation was defective in *evi5* Mo-injected tadpole limb. Scale bar represents 0.2 mm. **(L)** Stacking graph of non-regenerative, partial regenerative and full regenerative tadpole limbs.

Then, we examined the effect of knocking down Evi5 on *Xenopus* tadpole limb regeneration. As shown in Figure 3A, wound healing appeared normal after Evi5 knockdown by Mo; however, in *evi5* Mo-injected limbs the blastema formation was inhibited, and only a shrinking tip with a thin epithelium layer was formed at 3 dpa (as indicated with a black *, Figures 3B,C). HE staining of the limb stump sections showed that the wound epithelium in the *evi5* Mo-injected limb (Figure 3J) was not thickened like the wound epithelium in the *GFP* DNA injected limb, which also had a visible accumulation of blastema cells underneath the wound epithelium (Figure 3D–F,K). By 7 dpa, the GFP-injected limb had re-dedifferentiated digit-forming regions (Figure 3F), while the *evi5* Mo-injected limb formed a short epithelium-like tip (Figure 3C). This indicated that *evi5* Mo attenuated blastema formation. As a result, none of the *evi5* Mo-injected limb could fully regenerate, as indicated by the fewer digits formed in *evi5* Mo-injected limbs at 1 mpa (month after amputation), forming 0 to 2 digits in comparison of 3-4 digits in controls (Supplementary Figure S4). To confirm that the effect of *evi5* Mo on limb regeneration is specific, we co-injected *evi5* mRNA together with *evi5* Mo. The results showed that *evi5* mRNA could rescue about half of the injected limb to full regeneration (Figure 3L, Table 1).

**TABLE 1:** Summary of limb and tail regeneration after *evi5* Mo and mRNA injection in *Xenopus* tadpoles.

		Regeneration			N	χ^2^	*p*-value
		None	Partial	Full			
Tadpole limb	Mo	12	23	0	35	70.000	<0.001
	Control	0	0	35	35		
	Mo + mRNA	3	3	7	13		
Tadpole tail	Mo	9	4	4	17	19.319	<0.001
	Control	0	0	15	15		

Table notes: Regeneration of the limb and tail were determined by morphology of the regenerates described in material and method section. Chi-square test was used for significance analysis for Mo vs. Control.

Taken together, these observations demonstrated that Evi5 is required for *Xenopus* tadpole limb development and regeneration.

### Knockdown of Evi5 inhibits proliferation of *Xenopus* blastema cells

**FIGURE 4:**
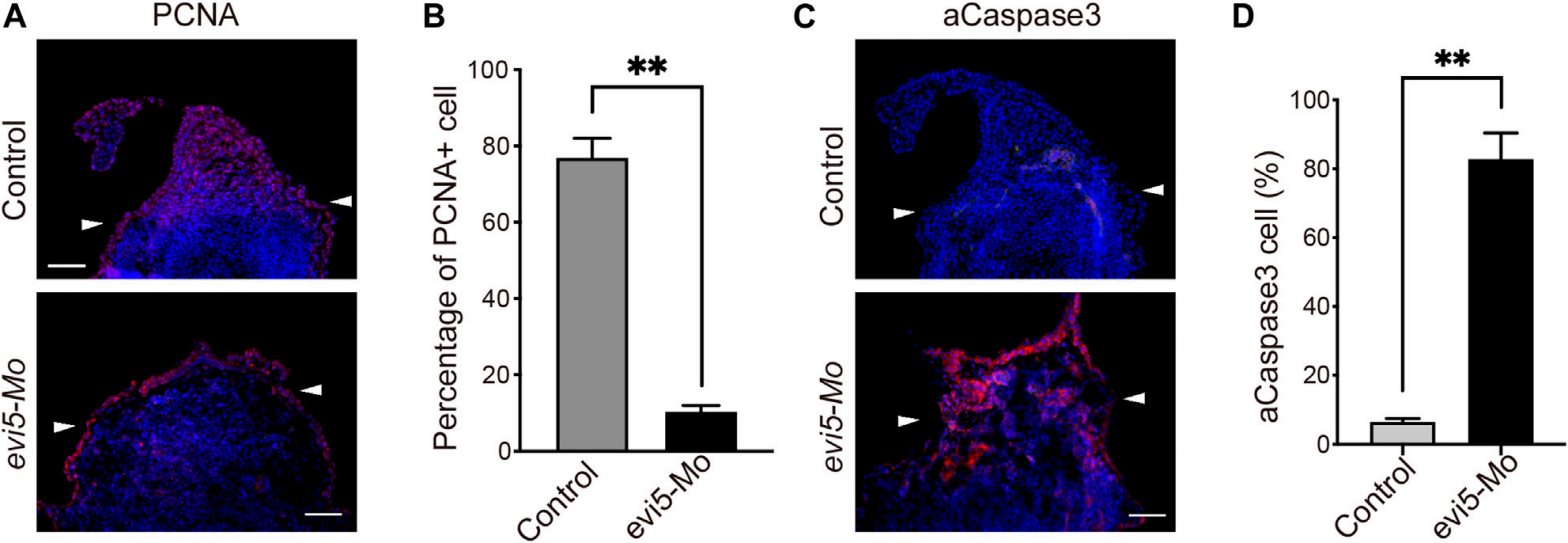
Proliferation and apoptosis of *Xenopus* tadpole limb blastema after Evi5 knockdown. **(A)** Immunofluorescence staining of PCNA (red) in *evi5* Mo-injected NF stage 52 tadpole hind limbs at 3 dpa. Nuclei were counterstained with DAPI, shown in blue. **(B)** Quantitative analysis of PCNA-positive cells in the limb stump mesenchyme, distal to the level marked by white arrowheads. ** indicates significant difference, *p* <0.01, *n* = 3, t-test. **(C)** Immunofluorescence staining of active Caspase3 (aCaspase3, shown in red) in *evi5* Mo-injected NF stage 52 tadpole hind limbs at 3 dpa. **(D)** Quantitative analysis of aCaspase3-positive cells in the limb stump distal to the level marked by white arrowheads. ** indicates significant difference, *p* <0.01, *n* = 3, t-test. Scale bars represent 100 μm.

The above observations showed that blastema formation is defective in the Evi5 knockdown tadpole limbs. This could be caused by the non-proliferation or death of progenitor cells in the limb stump. To address the effect of Evi5 knockdown on the proliferation of blastema cells, we performed PCNA immunofluorescence staining on the regenerating NF stage 52 tadpole limbs injected with *evi5* Mo. A large number of proliferating cells accumulated in the regenerating blastema during normal regeneration, but there was a lack of accumulation of proliferating cells after Evi5 knockdown (Figure 4A). Quantitative analysis revealed that the percentage of PCNA-positive nuclei in the Mo-injected group was significantly lower than that in the control group (Figure 4B). Evi5 was shown to regulate mitosis, and loss of function of Evi5 may lead to a mitotic catastrophe (Eldridge et al., 2006). We examined cellular apoptosis using immunofluorescence staining of active Caspase3 (aCaspase3). The results demonstrated that the proportion of aCaspase-positive cells after *evi5* Mo injection was significantly increased compared to the control, indicating that *evi5* Mo treatment induced cell apoptosis (Figures 4C,D).

The above *in vivo* experiment was performed on limb cells receiving Mo before a blastema was formed. To address whether Evi5 knockdown inhibits cell proliferation after the limb blastema cells have formed, we isolated blastema cells from the *Xenopus* tadpole limb regenerates and performed *in vitro* EdU incorporation analysis. This also confirmed that *evi5* Mo inhibited cell proliferation. As shown in Supplementary Figure S5A and Supplementary Figure S5B, about 45% of the control cells were EdU-positive, while almost none of the *evi5* Mo-transfected cells were EdU-positive, indicating that the down-regulation of Evi5 significantly inhibit proliferation of blastema cells.

### Evi5 is also required for tail regeneration

**FIGURE 5:**
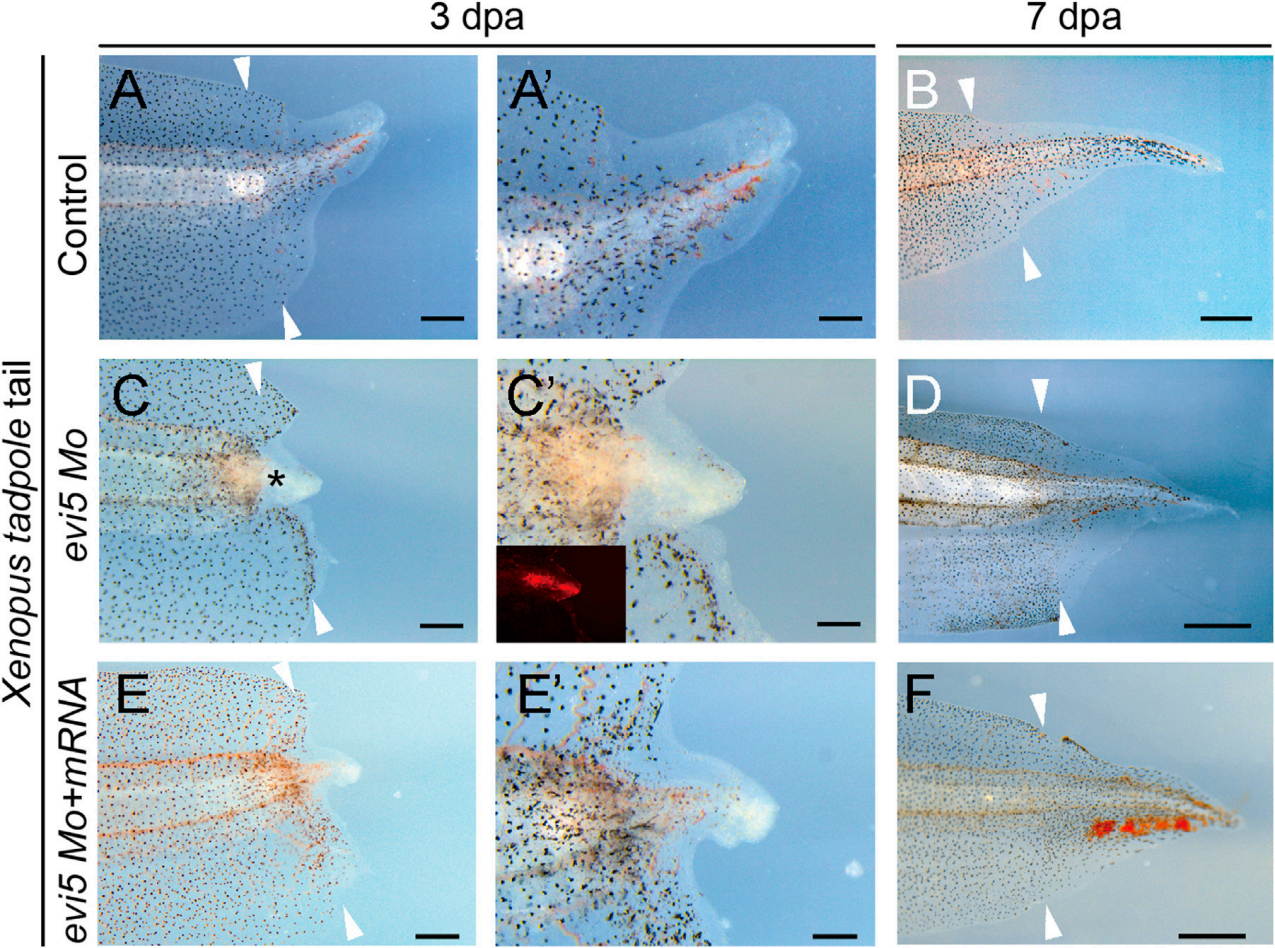
Tail regeneration in *Xenopus* tadpoles after *evi5* Mo injection. **(A–B)** Regenerating tadpole tail at 3 and 7 dpa in a control tadpole. **(C–D)** Regeneration of the Mo-injected tail was delayed compared to controls. * marks the tail blastema region lacking pigment cells. **(E–F)** Co-injection of *evi5* Mo and *evi5* mRNA partially rescued the inhibitory effect of *evi5* Mo on tail regeneration. **(A**’,**C**’,**E**’**)** High magnification images of tadpole tails at 3 dpa. Fluorescence signal in inset **(C’)** indicates the delivery of Mo. Scale bars represent 500 μm in **(A,C,E)**, 200 μm in **(A**’,**C**’,**E**’**)**, and 1 mm in **(B,D,F)**.

To address whether Evi5 is required generally for appendage regeneration, we used the tadpole tail as a regeneration model (Slack et al., 2008). Young *Xenopus* tadpoles can fully regenerate the tail, including the spinal cord, muscle, and pigment cells (Chen et al., 2006; Lin et al., 2007). We first detected *evi5* mRNA expression by RT-PCR and found that expression of *evi5* mRNA was upregulated after tail amputation, and reached a peak at 5 dpa (Figures 1D,E). Next, we injected and electroporated *evi5* Mo into the NF stage 48–49 *Xenopus* tadpole tails. The tails were amputated by a surgical blade 1 day after injection through the injection site, and the regeneration was observed at 3 and 7 dpa. The control tails had almost fully regenerated by 7 dpa (Figures 5A,B). At 3 dpa, most of the *evi5* Mo-injected tadpole tails showed defective regeneration, with smaller, and apparently less pigmented, blastema region (* in Figure 5C). We noted that about half of the *evi5* Mo-injected tadpole tails failed to regenerate, and half regained tail growth (Figure 5D; Table 1). This was probably related to the low efficiency of morpholino delivery into the tadpole tail region. Unlike the tadpole limb that could withhold the injected morpholino solution in its mesenchyme in a shape like a pocket (Supplementary Figure S3), it was harder for the tadpole tail to retain injected solution (as manifested by the inset in Figure 5C’). Nevertheless, the results showed that Evi5 protein is also required for *Xenopus* tail regeneration. And as in the case of tadpole limb amputation, co-injection of Mo and *evi5* mRNA also rescued the regeneration defect of the tadpole tail (Figures 5E–F, Table 1).

A similar expression pattern of *Evi*5 mRNA was found in axolotl tail regenerates. *Evi5* expression peaked at 5 dpa and returned to the control level at 14 dpa (Figure 1F). After electroporation of the *evi5* Mo into the axolotl tail, we observed that tail regeneration was inhibited (Supplementary Figure S6). This indicated that *evi5* transcription is required for both *Xenopus* and axolotl tail regeneration.

### RNA-seq analysis of *evi5* Mo-injected regenerating tadpole limbs

**FIGURE 6:**
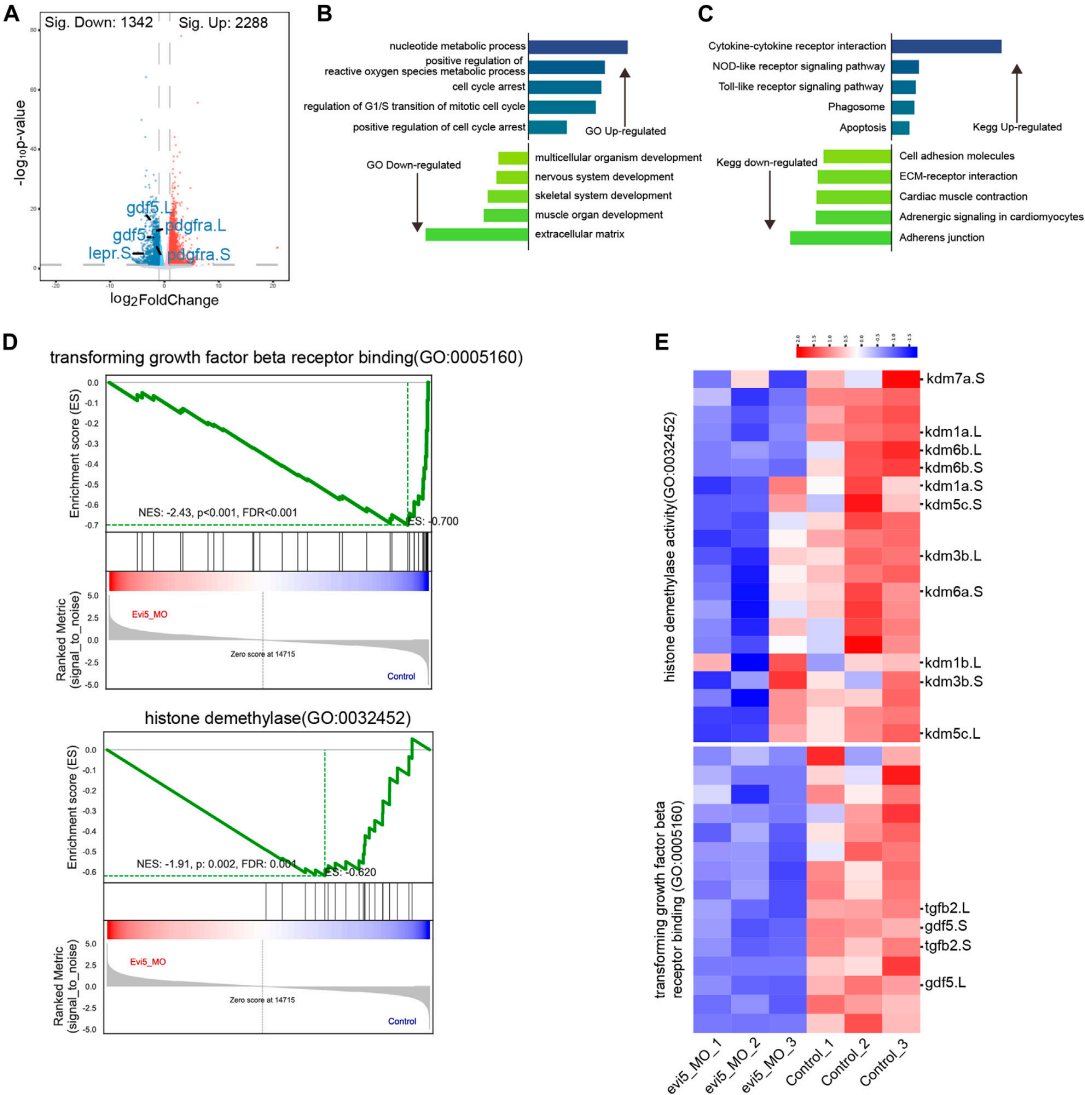
RNA-seq analysis of *evi5* Mo-injected *Xenopus* tadpole limbs at 3 dpa. **(A)** Volcano plot showing differential genes after Evi5 knockdown, with significant down-regulation markers in blue, significant up-regulation markers in red, and no significant difference markers in gray **(B)** GO enrichment analysis of the biological processes enriched by up- and down-regulated genes, histogram representation - Log10 (*p* value). **(C)** KEGG pathway enrichment analysis of pathways enriched by up- and down-regulated genes, histogram representation - Log10 (*p* value). **(D)** Gene set enrichment analysis (GSEA) of genes enrichment in histone demethylase activity and transforming growth factor beta receptor binding gene ontology. **(E)** Heatmap analysis of gene set for GSEA.

To gain insight into the mechanism of Evi5 in limb regeneration, we collected 3 dpa limb regenerates from control and *evi5* Mo-injected NF stage 52–53 tadpoles for RNA-seq analysis. We found that Evi5 knockdown altered the expression of 3,630 genes (FDR ≤0.05 and FC ≥ 2). There were 2,288 genes upregulated and 1,342 genes downregulated (Figure 6A). Examining the fold changes of individual genes, we found that the most downregulated genes after Evi5 knockdown include leptin receptor (*lepr*), growth and differentiation factor 5 (*gdf5*), and platelet-derived growth factor receptor (*pdgfr*) (Figure 6A). These factors have been shown to be important for tissue regeneration. For example, leptin signals play critical roles in driving regeneration in zebrafish, *Xenopus* and mouse organ regeneration (Love et al., 2011; Kang et al., 2016). In axolotl limb regeneration, platelet-derived growth factor signals induce fibroblast migration into the blastema (Currie et al., 2016), and *Pdgfr* is a marker for fibroblasts in limb and digit regeneration blastema (Johnston et al., 2016; Carr et al., 2019; Johnson et al., 2020).

GO and KEGG enrichment analysis showed that regulation of reactive oxygen species, cell cycle arrest, mitotic cell cycle regulation, and apoptosis were signification upregulated, while extracellular matrix, cell adhesion, and skeletal and muscle system development were downregulated (Figures 6B,C). The result is consistent to a requirement for Evi5 in preventing cells from the premature entry of mitosis, and loss of function of Evi5 may lead to mitotic catastrophe (i.e., a form of cell death due to aberrant mitosis) (Eldridge et al., 2006) resulting in failure of limb regeneration. This was also in agreement with our examination of the proliferation and cell death in the limb blastema region (Figure 4).

By gene set enrichment analysis (GSEA) (Subramanian et al., 2005), the transforming growth factor beta receptor binding gene ontology significantly downregulated (Figure 6D). The genes *tgfb2* and *gdf5* involved in the gene set for transforming growth factor beta receptor binding gene ontology were significantly downregulated by heatmap analysis (Figure 6E). The TGF-beta signaling pathway has been shown to be critical for the early phase of *Xenopus* appendage regeneration (Ho and Whitman, 2008; Nakamura et al., 2021), and has been extensively studied during axolotl limb regeneration, as reviewed in (Sader and Roy, 2022).

**FIGURE 7:**
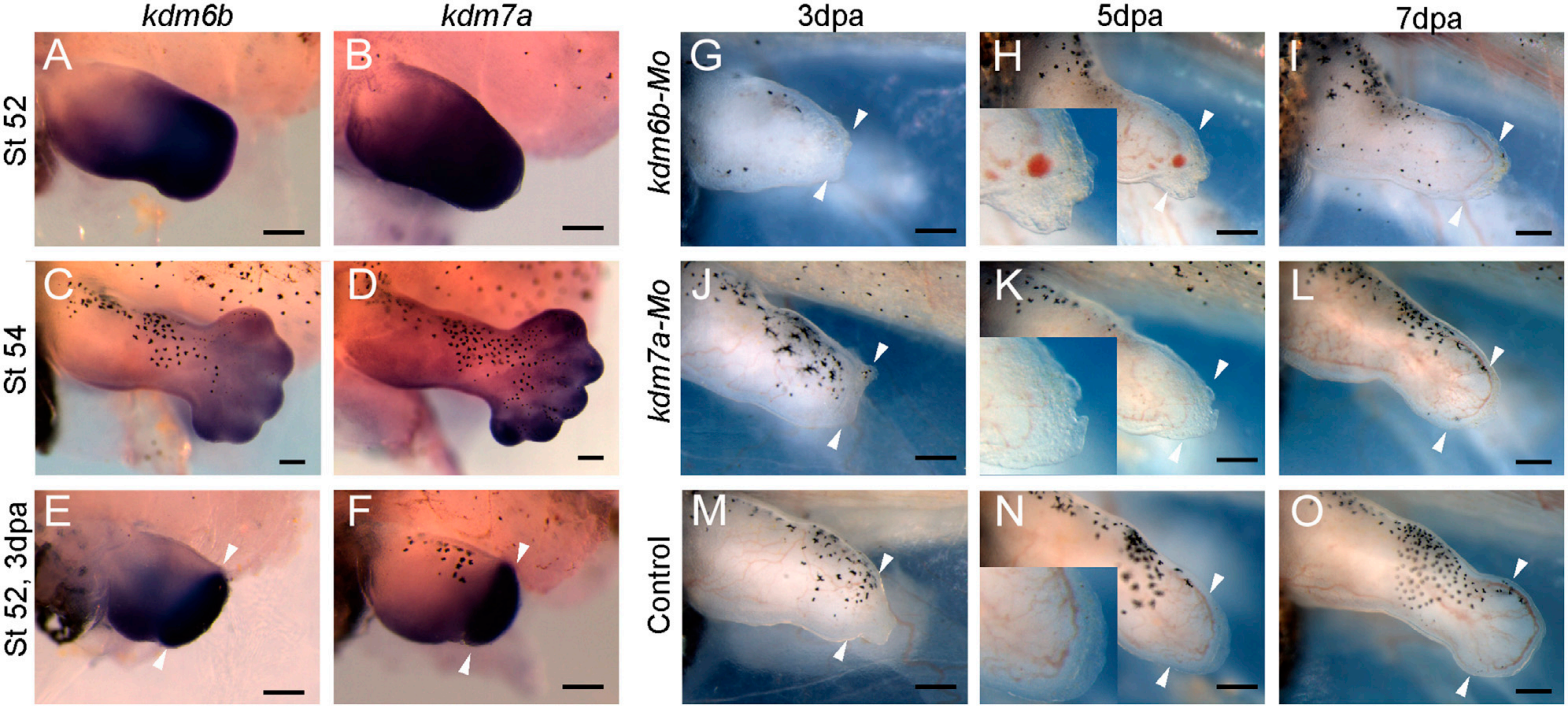
*kdm6b* and *kdm7a* in *Xenopus* tadpole hindlimb development and regeneration. **(A–F)** Expression of *kdm6b* **(A,C,E)** and *kdm7a* **(B,D,F)** in NF stage 52 **(A,B)** and stage 54 **(C,D)** *Xenopus* tadpole hindlimb, and 3 dpa limb regenerates (amputated at stage 52) **(E,F)**. **(G–O)** Representative images of tadpole hindlimbs at 3 dpa **(G,J,M)**, 5 dpa **(H,K,N)**, 7 dpa **(I,L,O)** after injection/electroporation of *kdm6b* Mo **(G–I)**, *kdm7a* Mo **(J–L)** or control Mo **(M–O)**. Images of tadpole limbs shown with dorsal up, anterior to the left. Scale bars represent 0.2 mm.

Interestingly, from our GSEA result, we observed that histone demethylase activity gene ontology was significantly downregulated (Figure 6D). By heatmap analysis of the gene set of histone demethylase activity, we found that the *kdm* genes were downregulated. Among them, lysine demethylase 6b and 7a (*kdm6b*, *kdm7a*) were significantly reduced in *evi5* Mo-injected tadpole hindlimb stumps (Figure 6E). Kdm6b has been shown to reactivate the expression of genes during zebrafish fin regeneration (Stewart et al., 2009). Thus, below we examined their expression during limb development and regeneration and showed that both *kdm6b* and *kdm7a* are required for *Xenopus* tadpole limb regeneration (Figure 7).

### Histone demethylase *kdm6b* and *kdm7a* as Evi5 targets in tadpole limb regeneration

The RNA-seq analysis identified that histone demethylase *kdm6b* and *kdm7a* were two genes significantly downregulated by Evi5 knockdown in the tadpole limbs (Figure 6E). By *in situ* hybridization, we confirmed that both *kdm6b* and *kdm7a* were highly expressed in NF stage 52–54 tadpole hindlimbs (Figures 7A–D). After limb amputation, *kdm6b* and *kdm7a* were strongly upregulated in the limb regenerates, especially in the blastema region (Figures 7E,F), suggesting that they regulate blastema formation in limb regeneration. We designed morpholinos against *kdm6b* or *kdm7a* and examined the effect of knocking down Kdm6b and Kdm7a on tadpole limb regeneration. As expected, knockdown of either *kdm6b* or *kdm7a* inhibited limb regeneration in the NF stage 52–53 tadpoles (Figures 7G–O). All tadpole hindlimbs healed the amputation wounds. However, in Kdm6b and Kdm7a knockdown tadpole hindlimbs, it was evident that the formation of limb blastema was defective. At 3 and 5 dpa, the Kdm6b and Kdm7a knockdown limb stumps were covered with a thin layer of epithelium (insets of Figures 7H,K). Thus, the current work identified *kdm6b* and *kdm7a* as downstream targets of Evi5 in *Xenopus* limb regeneration, placing histone demethylation as an important mechanism for further investigation.

## Discussion

### Expression and requirement of Evi5 for *Xenopus* limb and tail regeneration

Protein expression profiling of the axolotl limb began before the omics era (Slack, 1982, 1983), and continues to be a topic of great interest with the development of proteomic technologies (Rao et al., 2014; Sibai et al., 2020), with single-cell resolution (Gerber et al., 2018; Leigh et al., 2018). Evi5 was identified as one critical player in limb regeneration when Stocum lab compared the protein profile between the regenerating axolotl limb and the less regenerative froglet arm (Rao et al., 2014). Evi5 was significantly up-regulated in the regenerated hindlimbs of axolotl, but remained unchanged in *Xenopus* froglet forelimb amputated stump. This sharp contrast made Evi5 an interesting target for further investigating the molecular mechanisms underlying the declined regenerative capacity in the anuran amphibian *Xenopus* limbs. Our results reported here confirm that the expression of *evi5* at the mRNA level is significantly increased in tail and limb regeneration in regeneration-competent stages in *Xenopus* tadpoles (Figure 1). Evi5 is also functionally required for the regeneration of both the limb and the tail in *Xenopus* tadpoles (Figure 3, Figure 5).

We did attempt to overexpress *evi5* in NF stage 58 tadpoles and post-metamorphic froglets by injecting and electroporating overexpression plasmids or by using transgenic tadpoles overexpressing *evi5* under a heat shock inducible promoter, but it was not enough to promote hindlimb regeneration (Supplementary Figure S7). We also overexpressed *evi5* in the hindlimbs of NF stage 54–55 tadpoles that have reduced regenerative capacity compared to NF 52 stage tadpoles. The results showed that *evi5* significantly increased the area of the regenerated portion compared to the control, at 3 and 5 dpa, but *evi5* overexpression could not induce complete regeneration when examined at digit differentiation stages (Supplementary Figure S8). Nevertheless, we conclude that Evi5 is required, though being insufficient itself, for normal limb regeneration.

The quantitative PCR results, together with sectioning of the *in situ* hybridization specimens, showed that *evi5* is mainly expressed at the stages of formation and accumulation of blastema cells, and its expression level decreases when the blastema begins to differentiate and proliferate (Figure 1 and Figure 3). For example, in the regeneration of amputated limbs of NF stage 52 *Xenopus* tadpoles, *evi5* was highly expressed at 3 and 5 dpa, and gradually returned to normal levels at 7 dpa. Interestingly, levels of *evi5* mRNA decrease in post-metamorphic *Xenopus* forelimb after amputation (Figure 1). The cause of *evi5* downregulation is not clear, but may be related to the inability of full reprogramming of the fibroblastic blastema of *Xenopus* forelimb (Gerber et al., 2018).

While *evi5* mRNA could also be found in the wound epidermis (Figure 1), Evi5 knockdown mainly affected the formation of blastema, as there were extensive epithelial tissues still formed after *evi5* Mo injection (Figure 3). This may have been caused by the uneven distribution of *evi5* Mo to the blastemal mesenchyme by electroporation. Electroporation of DNA and oligonucleotides is widely used in amphibian regeneration studies, for example in the newt, axolotl, and *Xenopus* limb (Kumar et al., 2007; Sugiura et al., 2016; Zhang et al., 2018a). However, this method does have limitations. For example, the injected solutions need space for even distribution. In our hands, it is relatively easy to deliver DNA and morpholinos to *Xenopus* tadpole limb buds and axolotl tails, but it is much more difficult to deliver DNA and morpholinos to the *Xenopus* tadpole tail and axolotl limbs. This may well explain the less significant effect of *evi5* Mo on axolotl limb regeneration (not shown) and tadpole tail regeneration (Figure 5).

### Mechanisms of Evi5 in amphibian limb and tail regeneration

Based on previous and our current work, we argue that Evi5 regulates the process of appendage regeneration through multiple mechanisms. First, cell cycle biology studies showed that Evi5 accumulates in S/G2 phase by stably binding to the early division inhibitor Emi1, thereby arresting cells in G2 phase (Eldridge et al., 2006). During newt and axolotl limb regeneration, there are a large number of dedifferentiated cells and precursor cells re-entering the cell cycle, but these cells have a very low division index (Mescher and Tassava, 1975). Therefore, it was proposed that Evi5 may prevent the blastema cells from premature entry into division before a certain number of cells have accumulated (Rao et al., 2014). This process may also be required for proper reprogramming, dedifferentiation and re-specialization of the various cell types that constitute the blastema and the wound epithelium (Currie et al., 2016; Aztekin, 2021; Bassat and Tanaka, 2021; Lin et al., 2021). The reduced proliferation of *Xenopus* blastema cells (Figure 4) and dysregulation of cell cycle-related genes by Evi5 knockdown (Figure 6) indicate disruption of cell cycles. As a result, there was significant apoptosis in the limb mesenchyme after Evi5 knockdown (Figure 4).

Second, due to the Rab-GAP activity of Evi5, it can play a role in vesicle trafficking and endosome recycling, a mechanism that may help explain our observed lack of pigment cells in the tadpole tail regenerates (Figure 5). It has been shown that down-regulation of *Evi5* in drosophila ovary border cells interferes with border cell migration (Laflamme et al., 2012). The RNA-sequencing results indicated that knocking down Evi5 in the *Xenopus* tadpole limb may affect transport of substances and the binding of cytokines to receptors (Figure 6). We have utilized a skin punch assay on the froglet legs to investigate the migration of pigment cells after *evi5* Mo injection. This showed that there were severe delays of the healing process, with hampered pigment cell migration as exemplified by the reduced number of pigment cells in the skin wounds (Supplementary Figure S9). Thus, cell migration regulation is another target of Evi5 in wound healing and appendage regeneration.

In addition to the signals known to be important for regeneration, such as the TGFβ signals (Ho and Whitman, 2008; Nakamura et al., 2021), the reactive oxygen species (Love et al., 2013; Zhang et al., 2018a), it is interesting that the lysine demethylases *kdm6b* and *kdm7a* are among the most significantly down-regulated genes identified after Evi5 knockdown (Figures 6D,E). Properly controlled histone methylation and demethylation are important for appropriate gene expression for dedifferentiation of blastema cells (Hayashi et al., 2020). The methylation status of patterning gene, such as *shh*, has been linked to the regeneration capacity of the *Xenopus* limbs. In post-metamorphic *Xenopus* froglet limbs, the enhancer region of *shh* is hypermethylated, while in the regenerative *Xenopus* tadpoles, this region is demethylated (Yakushiji et al., 2007). However, the mechanisms responsible for the epigenetic control of regeneration genes have not been fully investigated.

*In situ* hybridization analysis of *kdm6b* and *kdm7a* showed that they are expressed in the developing limb and the regenerating limb blastema (Figure 7). Knockdown of Kdm6b and Kdm7a also caused a block in limb regeneration (Figures 7G–O). As has been demonstrated in appendage regeneration in other model animals, Kdm6b and Kdm7a may demethylate the promoter regions of genes important for regeneration so that the silenced genes are re-expressed, allowing dedifferentiation and proliferation of blastema cells. Evi5 as an upstream regulator of Kdm6b and Kdm7a has not been reported, so this finding provides a new possible mechanism for the action of Evi5 in appendage regeneration. Our ongoing investigation of how Evi5 regulates activities of Kdm6b and Kdm7a, and what are the downstream targets of Kdm6b and Kdm7a, shall shed light on our understanding of amphibian appendage regeneration.

## Methods

### Animal husbandry and microinjection

*Xenopus laevis* and *Ambystoma mexicanum* (axolotls) were obtained from in-house breeding. *Xenopus* embryos were procured by *in vitro* fertilization or natural mating, dejellied with 2% cysteine (pH 7.8, Sigma-Aldrich) and raised in 1/10 MMR (MMR, 100 mM NaCl, 2 mM KCl, 2 mM CaCl_2_, 1 mM MgSO_4,_ 5 mM HEPES, pH 7.4) (Sive et al., 2000). *Xenopus* and embryos were staged according to the Normal table of *Xenopus* *laevis* (Daudin) (Nieuwkoop and Faber, 1967). Axolotl embryos were obtained by the method of artificial fertilization as previously described (Mohun et al., 1980).

For *Xenopus* embryo microinjection, morpholino antisense oligos (Mo, 20 ng), or Mo and synthetic mRNAs (70 pg), were microinjected into one of the animal blastomeres of 4-8 cell stage embryos cultured in 2% Ficoll 400 in 0.4 × MMR, and raised in 1/10 MMR to tadpole stages.

Microinjection and electroporation in the tadpoles were performed as described (Zhang et al., 2018b) and also illustrated in Supplementary Figure S3. Briefly, animals of selected stages were anesthetized with 0.02% MS-222 (Sigma-Aldrich) and injected with control or *evi5* Mo, *evi5* mRNA, or pcDNA3-*GFP* DNA plasmid, immediately followed by electroporation, with a platinum Tweezertrodes electrodes (BTX, United States) attached to an ECM 830 square wave generator (BTX, United States). The gap between the electrodes was set as 2 mm, and the tadpoles were not directly touched by the electrodes. The setting of electroporation was voltage = 50 V/mm (for 2 mm gap electrodes, 100 V), pulse length = 10 ms, 1 pulse. Fluorescent signals were checked to confirm successful microinjection and electroporation. For tadpole tail electroporation, morpholino solutions were injected into the notochord areas, at the level of about 40% (the distal part) of the tail. The tail may require multiple injections, as it was harder for the tail tissue to retain the injected solution. Same setting of electroporation was used for the tadpole tail.

The next day after injection, the efficiency of electroporation was determined by examining the fluorescence signals (Supplementary Figure S3C), and limbs (or tails) were then amputated, as described below.

### Limb and tail amputation procedures

Animal experiments were carried out in compliance with the Association for Assessment and Accreditation of Laboratory Animal Care International (http://www.aaalac.org/index.cfm), and protocols were approved by the Institutional Animal Care and Use Committees (IACUC) of Tongji University.

For limb amputation, animals were anesthetized in 1/10 MMR containing 0.02% MS-222 (from stock of 0.5% MS-222, Sigma-Aldrich, United States, pH 7.5 buffered with Tris-Cl), NF stage 52–53 tadpole hindlimbs were amputated with a surgical scissor at the level of presumptive knee, and froglet forelimbs were amputated at mid-ulna/radius (Zhang et al., 2018a). For tail amputation, NF stage 49–51 tadpoles anesthetized were amputated with a surgical blade perpendicular to the notochord, removing 40% of the tail (Lin and Slack, 2008).

### DNA constructs and morpholino oligos

To obtain the sequence of *Xenopus laevis evi5* (*xl evi5*), primers *xl*-*evi5*-F: GTC​AGT​CAA​ATG​GCA​AGT​CAG​GTG​G and *xl*-*evi5*-R: ATT​AGC​AAT​CAC​AGT​AAC​CAT​CAA​A were designed based on the sequence information of *Xenopus tropicalis evi5*, before the *Xenopus laevis evi5* sequence was available. PCR fragment was amplified from cDNA samples of *Xenopus laevis* tadpole limb blastema, ligated with T4 ligase (NEB, United States) to pEasy-T1 vector (TransGen Biotech, China) and the sequence of the DNA plasmid was validated by Sanger sequencing (Sangon, Shanghai, China). Sequencing result showed that the full-length *Xlevi5* is highly homologous to both *Xenopus laevis evi5.L* (including XM_018258571.2) and *S* (XM_041561525.1), though the original *xl*-*evi5*-R primer used has 3 mismatched bases in the sequence after the stop codon.

Primers used for obtaining *kdm6b* probe construct: F: ATG​AAG​GTT​CCG​GGC​AGC​AG, R: TCA​CCG​GAT​GTT​CGG​GGG​TGG; Primers used for *kdm7a* probe construct: F: ATG​GCC​GGA​GCG​GCT​CCA​GTG​TA, R: TTA​AAC​CAT​AAA​ATA​ACC​AAG​GTT​CGC​TC. These probe constructs were designed based on *kdm6b.S* and *kdm7a.S*.

For probe synthesis, constructs were linearized and transcribed with T7 or T3 RNA polymerase with DIG RNA labeling mix (Roche). *evi5* mRNA was prepared with mMessenger mMachine kit (Ambion, United States).

Morpholino antisense oligos were designed and synthesized by Gene Tools Inc. The sequences were: *evi5* Mo: CCA​CCT​GAC​TTG​CCA​TTT​GAC​TGA​C; *kdm6b* Mo: CTG​TGG​GCG​ATA​CAT​CCA​GCC​G; *kdm7a* Mo: CGC​TCC​GGC​CAT​CTT​TAA​ATC​CCA​C. Standard control: CCT​CTT​ACC​TCA​GTT​ACA​ATT​TAT​A. The *evi5* Mo blocks both *evi5.L* and *evi5.S*; *kdm6b* Mo targets *kdm6b.S*, with one base mismatch for *kdm6b.L*; *kdm7a* Mo targets *kdm7a.S*.

### RT-PCR

Total RNA was extracted using TRIzol reagent (Invitrogen) according to the manufacturer’s instructions. RNA samples were subsequently treated with DNaseI (Invitrogen) before being reverse transcribed into cDNAs with the Superscript III Reverse Transcriptase system (Invitrogen). The cDNAs were then used for real-time PCR, which contained the fluorescent dye SYBR Green (Sigma-Aldrich) to monitor DNA synthesis. Primers used were: *xl-ef1a*: F 5'-CCT​GAA​CCA​CCC​AGG​CCA​GAT​TGG​TG-3', R 5'-GAG​GGT​AGT​CAG​AGA​AGC​TCT​CCA​CG-3'; *xl*-*evi5*: F 5'-AGG​AGG​TGA​TGG​CAG​TTC​GG-3', R 5'-AGT​GGG​TTG​GTC​TGG​GAG​GC-3'; *Am*-*Rps21*: F 5'-ACT​TGA​AGT​TTG​TTG​CCA​GGA​C-3', R 5'-TGG​CAT​CTT​CTA​TGA​TCC​CAT​C-3'; *Am*-*Evi5*: F 5'-GTTCTTCAGCATCCA GCAATCTC-3'; R 5'-CTT​TCT​TCT​TGC​GTG​CAT​CTT​CC-3'. *ef1a* and *Rps21* were used as internal references.

### *In situ* hybridization

Whole-mount *in situ* hybridization (WISH) was performed for *evi5*, *kdm6b* and *kdm7a* mRNA detection, following standard protocols (Sive et al., 2000). Samples collected at desired time points were fixed with MEMFA fixative (0.1 M MOPS, 2 mM EGTA, 1 mM MgSO_4_, 4% PFA, pH 7.4). *In situ* hybridization of advanced staged tadpole limbs was performed with modifications as previously described (Lin et al., 2013).

### Western blotting

Limbs were harvested and lysed in RIPA buffer (ThermoFischer Scientific) supplemented with complete protease inhibitors (Roche). Protein concentration for each lysate was measured using a BCA protein assay kit (ThermoFischer Scientific). Proteins were separated by electrophoresis on SDS-polyacrylamide gels and subsequently processed for standard Western blotting. The primary antibody (anti-Evi5, ab70790) and HRP-conjugated secondary antibodies (Invitrogen) were diluted using 5% (w/v) skimmed milk in TBST (20 mM Tris, 150 mM NaCl, 0.1% (w/v) Tween 20). Immunoreactive signals were detected using ECL substrate (Tanon, Cat#180–501) and imaged with an Amersham Imager 600 imaging system (GE Healthcare).

### Histology

Tissues fixed in 4% PFA were processed for paraffin (ThermoFisher) embedding and then sectioned at 7 μm with an LM2016 microtome (Leica Biosystems). Hematoxylin and eosin (HE) staining was performed on paraffin sections according to standard protocol. For histology analysis of *in situ* hybridization specimens, samples were refixed briefly and sectioned, dewaxed, and then processed without staining. Slides were mounted in Permount mounting medium (Fisher Scientific) before observation.

### RNA-sequencing and analysis

Total RNA was extracted from the limb stumps using TRIzol Reagent (Invitrogen) and genomic DNA was removed using DNase I (TaKara). RNA purity and quantification were evaluated using the NanoDrop 2000 spectrophotometer (Thermo Scientific, United States). RNA integrity was assessed using the Agilent 2,100 Bioanalyzer (Agilent Technologies, Santa Clara, CA, United States). Then the libraries were constructed using VAHTS Universal V6 RNA-seq Library Prep Kit according to the manufacturer’s instructions. Illumina Novaseq 6,000 platform was applied for transcriptome sequencing, conducted by OE Biotech Co., Ltd. (Shanghai, China). Raw reads of fastq format were firstly processed using fastp and the low-quality reads were removed to obtain the clean reads for subsequent analyses. The clean reads were mapped to the reference genome XENLA_10.1 (available at https://ftp.xenbase.org/pub/Genomics/JGI/Xenla10.1/XENLA_10.1_genome.fa.gz) using HISAT2. Using htseq-count software and annotation files XENLA_10.1_GCF.gff3 (available at https://ftp.xenbase.org/pub/Genomics/JGI/Xenla10.1/XENLA_10.1_GCF.gff3) to obtain the count of gene reads in each sample. Differentially expressed genes (DEGs) were identified using the R statistical package edgeR (Empirical Analysis of Digital Gene Expression in R), employing a threshold of false discovery rate (FDR) ≤ 0.05 and fold change (FC) ≥ 2. GO and KEGG pathway enrichment analyses were used to obtain the functional annotation of up- and down-regulated genes. Gene Set Enrichment Analysis (GSEA) software was obtained from http://www.gsea-msigdb.org and the gene set for analysis was obtained from http://geneontology.org.

### Microscopy and photography

Regeneration and fluorescent protein expression in live embryos or animals under anesthetic were observed using a Leica M165FC fluorescent dissecting microscope with eGFP and RFP filter sets. Slides were observed using a Leica DM6000B inverted microscope. Images were captured using a Leica camera and processed with Photoshop software (Adobe).

### Regeneration quantitation and statistical analysis

Tail regeneration was classified as full, partial or non-regeneration as previously described (Beck et al., 2003). For limb regeneration, regenerates with patterned digit formation were measured as full regeneration, and regenerates with less than 2 digit forming regions were counted as partial regeneration. Limb stump with epithelium but no elongation of underlying blastema region was considered nonregenerative. Chi-square was used for statistical analysis between Mo-injected and control regeneration, as shown in Table 1. Percentages of PCNA and aCaspase3 positive cells were compared with *t*-tests between groups. Data were presented as mean +/- standard deviation. Differences were considered significant if the *p*-value <0.05(*) or <0.01(**).

## Data Availability

The data presented in the study are deposited in the GEO repository, accession number GSE218034.

## References

[B1] Aztekin C. (2021). Tissues and cell types of appendage regeneration: A detailed look at the wound epidermis and its specialized forms. Front. Physiol. 12, 771040. 10.3389/fphys.2021.771040 34887777 PMC8649801

[B2] Bassat E. Tanaka E. M. (2021). The cellular and signaling dynamics of salamander limb regeneration. Curr. Opin. Cell Biol. 73, 117–123. 10.1016/j.ceb.2021.07.010 34521022

[B3] Beck C. W. Christen B. Slack J. M. W. (2003). Molecular pathways needed for regeneration of spinal cord and muscle in a vertebrate. Dev. Cell 5 (3), 429–439. 10.1016/s1534-5807(03)00233-8 12967562

[B4] Carlson B. M. (2007). Principles of regenerative biology. Burlington MA: Academic Press.

[B5] Carr M. J. Toma J. S. Johnston A. P. W. Steadman P. E. Yuzwa S. A. Mahmud N. (2019). Mesenchymal precursor cells in adult nerves contribute to mammalian tissue repair and regeneration. Cell Stem Cell 24 (2), 240–256. 10.1016/j.stem.2018.10.024 30503141

[B6] Chen Y. Lin G. Slack J. M. W. (2006). Control of muscle regeneration in the Xenopus tadpole tail by Pax7. Development 133 (12), 2303–2313. 10.1242/dev.02397 16687446

[B7] Cox B. D. Yun M. H. Poss K. D. (2019). Can laboratory model systems instruct human limb regeneration? Development 146 (20), dev181016. 10.1242/dev.181016 31578190 PMC6917474

[B8] Currie J. D. Kawaguchi A. Traspas R. M. Schuez M. Chara O. Tanaka E. M. (2016). Live imaging of axolotl digit regeneration reveals spatiotemporal choreography of diverse connective tissue progenitor pools. Dev. Cell 39 (4), 411–423. 10.1016/j.devcel.2016.10.013 27840105 PMC5127896

[B9] Davidian D. Levin M. (2022). Inducing vertebrate limb regeneration: A review of past advances and future outlook. Cold Spring Harb. Perspect. Biol. 14 (4), a040782. 10.1101/cshperspect.a040782 34400551 PMC9121900

[B10] Dent J. N. (1962). Limb regeneration in larvae and metamorphosing individuals of the South African clawed toad. J. Morphol. 110, 61–77. 10.1002/jmor.1051100105 13885494

[B11] Eldridge A. G. Loktev A. V. Hansen D. V. Verschuren E. W. Reimann J. D. Jackson P. K. (2006). The evi5 oncogene regulates cyclin accumulation by stabilizing the anaphase-promoting complex inhibitor emi1. Cell 124 (2), 367–380. 10.1016/j.cell.2005.10.038 16439210

[B12] Gerber T. Murawala P. Knapp D. Masselink W. Schuez M. Hermann S. (2018). Single-cell analysis uncovers convergence of cell identities during axolotl limb regeneration. Science 362 (6413), eaaq0681. 10.1126/science.aaq0681 30262634 PMC6669047

[B13] Gray P. A. Fu H. Luo P. Zhao Q. Yu J. Ferrari A. (2004). Mouse brain organization revealed through direct genome-scale TF expression analysis. Science 306 (5705), 2255–2257. 10.1126/science.1104935 15618518

[B14] Hayashi S. Tamura K. Yokoyama H. (2020). Chromatin dynamics underlying the precise regeneration of a vertebrate limb - epigenetic regulation and cellular memory. Semin. Cell Dev. Biol. 97, 16–25. 10.1016/j.semcdb.2019.04.006 30991117

[B15] Ho D. M. Whitman M. (2008). TGF-β signaling is required for multiple processes during Xenopus tail regeneration. Dev. Biol. 315 (1), 203–216. 10.1016/j.ydbio.2007.12.031 18234181 PMC2292344

[B16] Johnson G. L. Masias E. J. Lehoczky J. A. (2020). Cellular heterogeneity and lineage restriction during mouse digit tip regeneration at single-cell resolution. Dev. Cell 52 (4), 525–540. 10.1016/j.devcel.2020.01.026 32097654 PMC7186907

[B17] Johnston A. P. Yuzwa S. A. Carr M. J. Mahmud N. Storer M. A. Krause M. P. (2016). Dedifferentiated schwann cell precursors secreting paracrine factors are required for regeneration of the mammalian digit tip. Cell Stem Cell 19 (4), 433–448. 10.1016/j.stem.2016.06.002 27376984

[B18] Kang J. Hu J. Karra R. Dickson A. L. Tornini V. A. Nachtrab G. (2016). Modulation of tissue repair by regeneration enhancer elements. Nature 532 (7598), 201–206. 10.1038/nature17644 27049946 PMC4844022

[B19] Kumar A. Godwin J. W. Gates P. B. Garza-Garcia A. A. Brockes J. P. (2007). Molecular basis for the nerve dependence of limb regeneration in an adult vertebrate. Sci. (New York, NY) 318 (5851), 772–777. 10.1126/science.1147710 PMC2696928 17975060

[B20] Laflamme C. Assaker G. Ramel D. Dorn J. F. She D. Maddox P. S. (2012). Evi5 promotes collective cell migration through its Rab-GAP activity. J. Cell Biol. 198 (1), 57–67. 10.1083/jcb.201112114 22778279 PMC3392932

[B21] Leigh N. D. Dunlap G. S. Johnson K. Mariano R. Oshiro R. Wong A. Y. (2018). Transcriptomic landscape of the blastema niche in regenerating adult axolotl limbs at single-cell resolution. Nat. Commun. 9 (1), 5153. 10.1038/s41467-018-07604-0 30514844 PMC6279788

[B22] Lim Y. S. Tang B. L. (2013). The Evi5 family in cellular physiology and pathology. FEBS Lett. 587 (12), 1703–1710. 10.1016/j.febslet.2013.04.036 23669355

[B23] Lin G. Chen Y. Slack J. M. (2013). Imparting regenerative capacity to limbs by progenitor cell transplantation. Dev. Cell 24 (1), 41–51. 10.1016/j.devcel.2012.11.017 23273877 PMC3549047

[B24] Lin G. Chen Y. Slack J. M. W. (2007). Regeneration of neural crest derivatives in the Xenopus tadpole tail. BMC Dev. Biol. 7, 56. 10.1186/1471-213X-7-56 17521450 PMC1890292

[B25] Lin G. Slack J. M. W. (2008). Requirement for Wnt and FGF signaling in Xenopus tadpole tail regeneration. Dev. Biol. 316 (2), 323–335. 10.1016/j.ydbio.2008.01.032 18329638

[B26] Lin T. Y. Gerber T. Taniguchi-Sugiura Y. Murawala P. Hermann S. Grosser L. (2021). Fibroblast dedifferentiation as a determinant of successful regeneration. Dev. Cell 56 (10), 1541–1551. 10.1016/j.devcel.2021.04.016 34004152 PMC8140481

[B27] Love N. R. Chen Y. Bonev B. Gilchrist M. J. Fairclough L. Lea R. (2011). Genome-wide analysis of gene expression during Xenopus tropicalis tadpole tail regeneration. BMC Dev. Biol. 11, 70. 10.1186/1471-213X-11-70 22085734 PMC3247858

[B28] Love N. R. Chen Y. Ishibashi S. Kritsiligkou P. Lea R. Koh Y. (2013). Amputation-induced reactive oxygen species are required for successful Xenopus tadpole tail regeneration. Nat. Cell Biol. 15 (2), 222–228. 10.1038/ncb2659 23314862 PMC3728553

[B29] Mescher A. L. Tassava R. A. (1975). Denervation effects on DNA replication and mitosis during the initiation of limb regeneration in adult newts. Dev. Biol. 44 (1), 187–197. 10.1016/0012-1606(75)90386-3 1132586

[B30] Mohun T. J. Tilly R. Mohun R. Slack J. M. W. (1980). Cell commitment and gene expression in the axolotl embryo. Cell 22 (1), 9–15. 10.1016/0092-8674(80)90149-X 6893572

[B31] Nakamura M. Yoshida H. Moriyama Y. Kawakita I. Wlizla M. Takebayashi-Suzuki K. (2021). TGF-β1 signaling is essential for tissue regeneration in the Xenopus tadpole tail. Biochem. Biophys. Res. Commun. 565, 91–96. 10.1016/j.bbrc.2021.05.082 34102475 PMC8255271

[B32] Nieuwkoop P. D. Faber J. (1967). Normal table of *Xenopus laevis* (daudin). Amsterdam: North-Holland.

[B33] Rao N. Jhamb D. Milner D. J. Li B. Song F. Wang M. (2009). Proteomic analysis of blastema formation in regenerating axolotl limbs. BMC Biol. 7, 83. 10.1186/1741-7007-7-83 19948009 PMC2794268

[B34] Rao N. Song F. Jhamb D. Wang M. Milner D. J. Price N. M. (2014). Proteomic analysis of fibroblastema formation in regenerating hind limbs of *Xenopus laevis* froglets and comparison to axolotl. BMC Dev. Biol. 14, 32. 10.1186/1471-213x-14-32 25063185 PMC4222900

[B35] Reimann J. D. Freed E. Hsu J. Y. Kramer E. R. Peters J. M. Jackson P. K. (2001). Emi1 is a mitotic regulator that interacts with Cdc20 and inhibits the anaphase promoting complex. Cell 105 (5), 645–655. 10.1016/s0092-8674(01)00361-0 11389834

[B36] Sader F. Roy S. (2022). Tgf-beta superfamily and limb regeneration: Tgf-beta to start and Bmp to end. Dev. Dyn. 251 (6), 973–987. 10.1002/dvdy.379 34096672

[B37] Satoh A. Endo T. Abe M. Yakushiji N. Ohgo S. Tamura K. (2006). Characterization of Xenopus digits and regenerated limbs of the froglet. Dev. Dyn. 235 (12), 3316–3326. 10.1002/dvdy.20985 17075873

[B38] Sibai M. Altuntas E. Suzek B. E. Sahin B. Parlayan C. Ozturk G. (2020). Comparison of protein expression profile of limb regeneration between neotenic and metamorphic axolotl. Biochem. Biophys. Res. Commun. 522 (2), 428–434. 10.1016/j.bbrc.2019.11.118 31767146

[B39] Simon A. Tanaka E. M. (2013). Limb regeneration. Wiley Interdiscip. Rev. Dev. Biol. 2 (2), 291–300. 10.1002/wdev.73 24009038

[B40] Sive H. L. Grainger R. M. Harland R. M. (2000). Early development of *Xenopus laevis*: A laboratory manual cold spring harbor. New York: Cold Spring Harbor Laboratory Press.

[B41] Slack J. M. Lin G. Chen Y. (2008). The Xenopus tadpole: A new model for regeneration research. Cell. Mol. Life Sci. 65 (1), 54–63. 10.1007/s00018-007-7431-1 18030419 PMC11131608

[B42] Slack J. M. (1982). Protein synthesis during limb regeneration in the axolotl. Development 70, 241–260. 10.1242/dev.70.1.241 7142900

[B43] Slack J. M. (1983). Regional differences of protein synthesis in the limb regeneration blastema of the axolotl. Prog. Clin. Biol. Res. 110, 557–563. 6828516

[B44] Stewart S. Tsun Z. Y. Izpisua Belmonte J. C. (2009). A histone demethylase is necessary for regeneration in zebrafish. Proc. Natl. Acad. Sci. U. S. A. 106 (47), 19889–19894. 10.1073/pnas.0904132106 19897725 PMC2785262

[B45] Stocum D. L. Cameron J. A. (2011). Looking proximally and distally: 100 years of limb regeneration and beyond. Dev. Dyn. 240 (5), 943–968. 10.1002/dvdy.22553 21290477

[B46] Subramanian A. Tamayo P. Mootha V. K. Mukherjee S. Ebert B. L. Gillette M. A. (2005). Gene set enrichment analysis: A knowledge-based approach for interpreting genome-wide expression profiles. Proc. Natl. Acad. Sci. U. S. A. 102 (43), 15545–15550. 10.1073/pnas.0506580102 16199517 PMC1239896

[B47] Sugiura T. Wang H. Barsacchi R. Simon A. Tanaka E. M. (2016). MARCKS-like protein is an initiating molecule in axolotl appendage regeneration. Nature 531 (7593), 237–240. 10.1038/nature16974 26934225 PMC4795554

[B48] Yakushiji N. Suzuki M. Satoh A. Sagai T. Shiroishi T. Kobayashi H. (2007). Correlation between Shh expression and DNA methylation status of the limb-specific Shh enhancer region during limb regeneration in amphibians. Dev. Biol. 312 (1), 171–182. 10.1016/j.ydbio.2007.09.022 17961537

[B49] Zhang M. Chen Y. Xu H. Yang L. Yuan F. Li L. (2018a). Melanocortin receptor 4 signaling regulates vertebrate limb regeneration. Dev. Cell 46 (4), 397–409. 10.1016/j.devcel.2018.07.021 30130530 PMC6107305

[B50] Zhang M. Yang L. Yuan F. Chen Y. Lin G. (2018b). Dicer inactivation stimulates limb regeneration ability in *Xenopus laevis* . Wound Repair Regen. 26 (1), 46–53. 10.1111/wrr.12619 29453851

