# Supplementary material for: Evi5 is required for Xenopus limb and tail regeneration
Source: Front Cell Dev Biol. 2022 Dec 8;10:1027666. doi: 10.3389/fcell.2022.1027666 (PMC9809974; doi:10.3389/fcell.2022.1027666)
Supplement: Supplementary file 1 [file DataSheet1.pdf]

Supplementary Information

Evi5 is required for *Xenopus* limb and tail regeneration

Li Yang[1,#], Youwei Chen[1,#], Huahua Liu[1], Yu Liu[1], Feng Yuan[1], Qianyan Li[1] and Gufa Lin[1,*]

1Key Laboratory of Spine and Spinal Cord Injury Repair and Regeneration of Ministry of Education, Orthopaedic Department of Tongji Hospital, School of Life Sciences and Technology, Tongji University, Shanghai, China

*Correspondence: lingufa@tongji.edu.cn (G.L)

[#] These authors contributed equally to this work.

Supplementary Figures

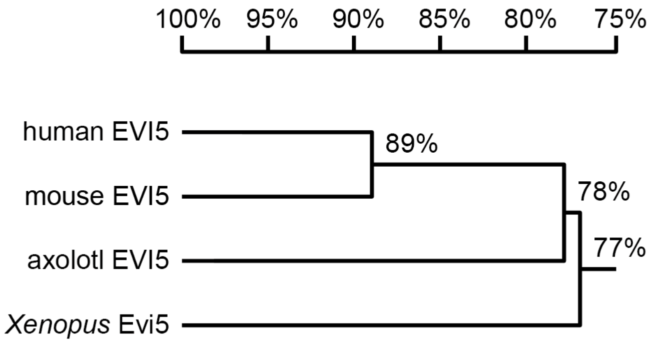

Figure S1. Sequence comparison of Evi5. Comparison of human, mouse, axolotl and *Xenopus* Evi5 protein sequence.

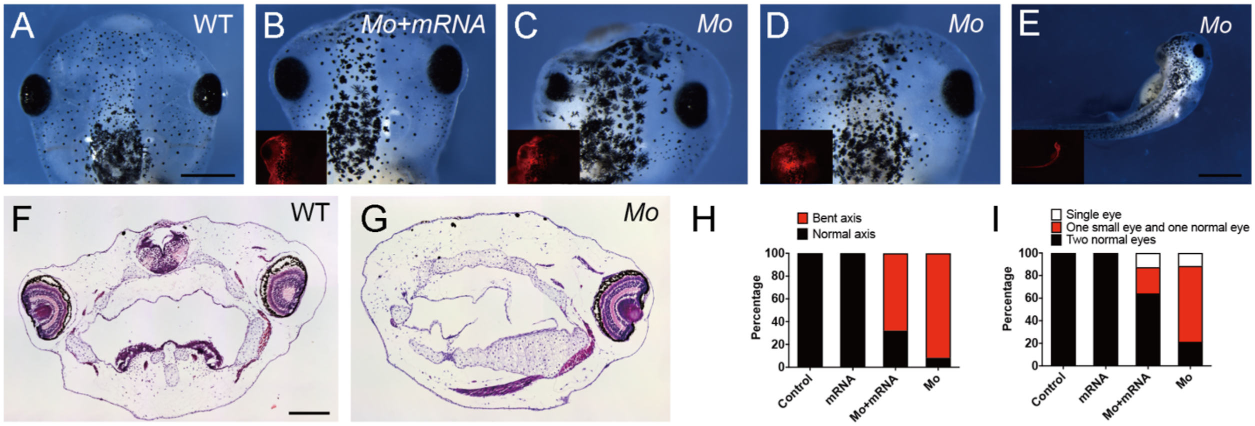

Figure S2. Effect of *evi5* Mo on embryonic development of *Xenopus*. (A-E) Knockdown of Evi5 expression during embryonic development, resulting in a smaller eye on the injected side (**C**) and even an eyeless phenotype (**D**). Co-injection of *evi5* Mo with *evi5* mRNA partially rescues the abnormal eye development (**B**). Injection of *evi5* Mo also causes body axis bending (**E**). **(F, G)** HE staining of wild-type tadpoles (**F**) and Mo-injected tadpoles (**G**). **(H, I)** Statistics of body axis bending phenotype and small eye phenotype in each injection group. Scale bars represent 1 mm in (**E**), 500 μm in (**A-D**), and 200 μm in (**F, G**).

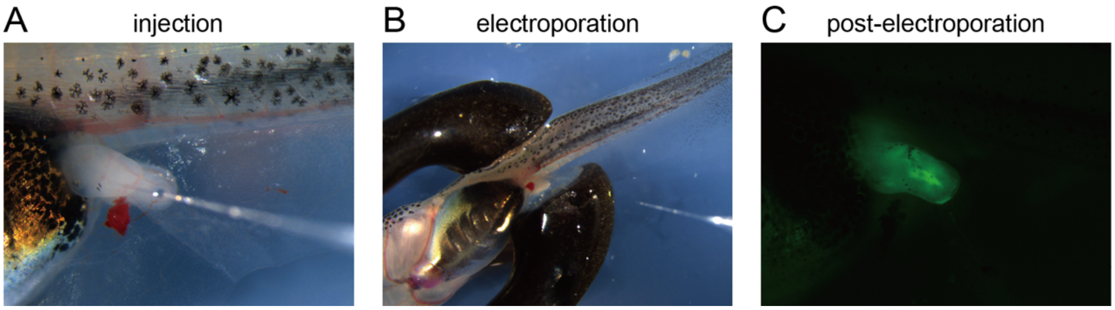

Figure S3. **Injection and electroporation method**. **(A)** Injection of Mo into the hind limb of NF stage 52 tadpoles using a pulled glass micropipette. **(B)** Tweezer-style electrodes placed on both sides of the limb for electroporation. **(C)** Fluorescence detection after electroporation.

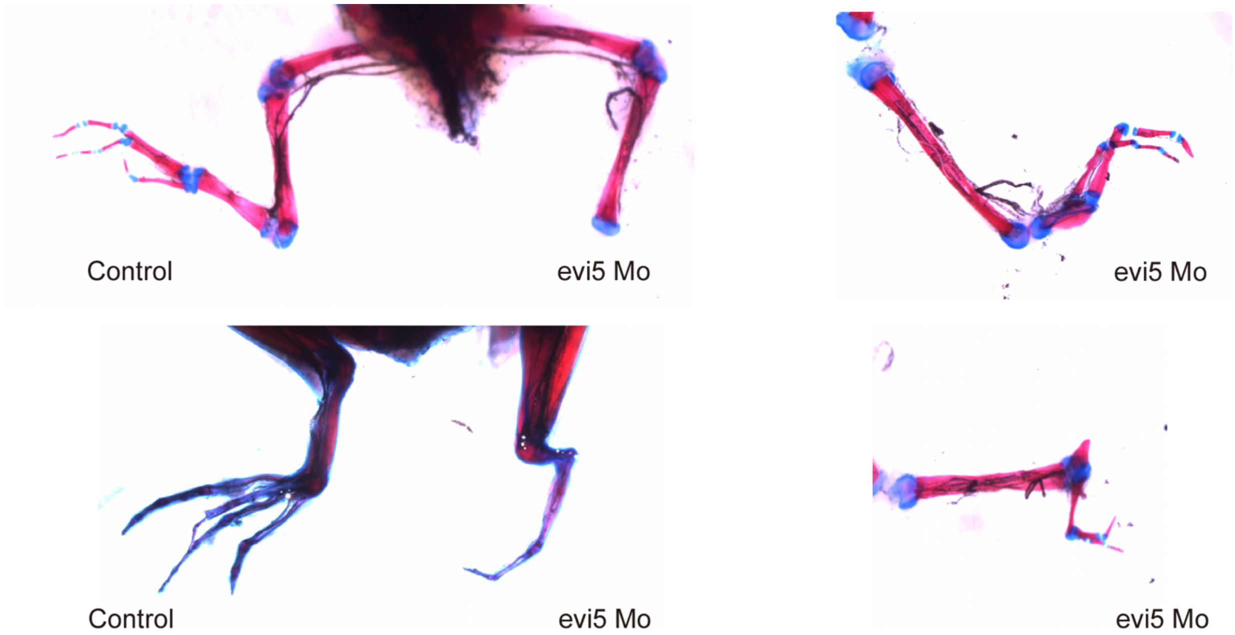

Figure S4. **Skeletal preparation of Evi5 knockdown tadpoles**. Shown are examples of skeletal staining of the hindlimbs of *Xenopus* froglets, 1 month after Evi5 knockdown at NF stage 52/53. While hindlimbs injected and electroporated with Control Mo regenerate 3-4 digits, hindlimbs with Evi5 knockdown regenerate fewer digits, forming 0 to 2 digits. Stage 52/53 tadpole hindlimbs were amputated through the knee level.

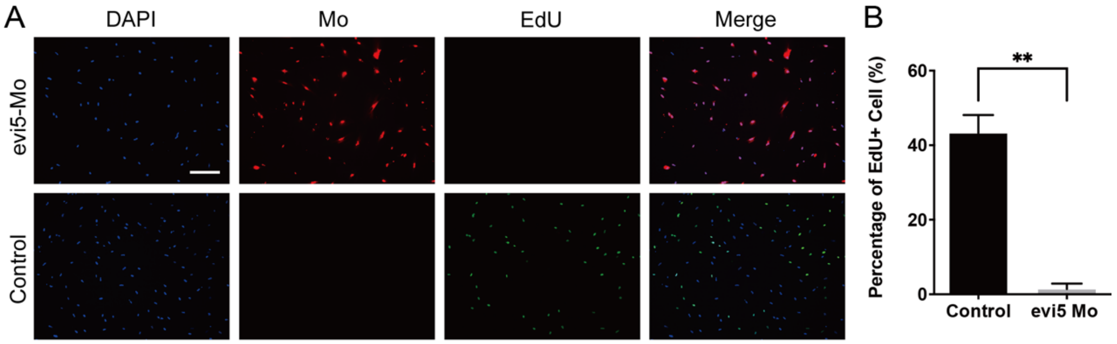

Figure S5. Proliferation of *Xenopus* tadpole limb blastema cells after *evi5* knockdown. (A) Micrographs of *Xenopus* blastema cells, after EdU incorporation detection. Mo was indicated by red fluorescence. EdU shown in green. Nuclei were counterstained with DAPI, shown in blue. Scale bar represents 200 µm. **(B)** EdU-positive cells/Mo-transfected cells. ** indicates significant difference, p<0.01, n=6, t-test.

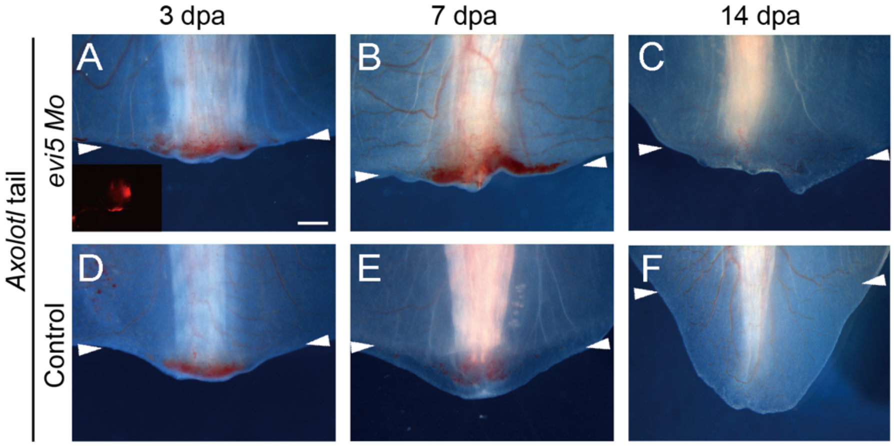

Figure S6. Axolotl tail regeneration after Evi5 knockdown. (A-C) Axolotl tail regeneration was inhibited by *evi5* Mo-injection. **(D-F)** Axolotl tail regeneration occurred normally in controls. White arrowheads indicate amputation level. Scale bar: 1mm.

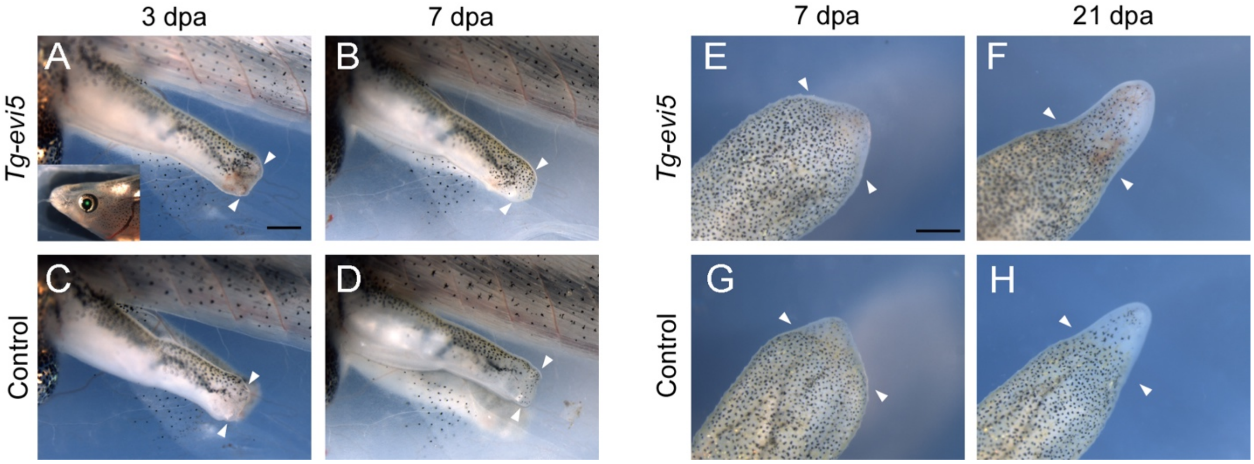

Figure S7. Overexpression of *evi5* in transgenic tadpoles and post-metamorphic froglets. (A-D) Continuous induction of *evi5* expression in transgenic tadpoles of NF stage 58 and observation of hind limb regeneration showed that systemic *evi5* overexpression did not promote regeneration. **(E-H)** Overexpression of *evi5* in post-metamorphic froglets did not facilitate limb regeneration. Scale bar: 500 µm.

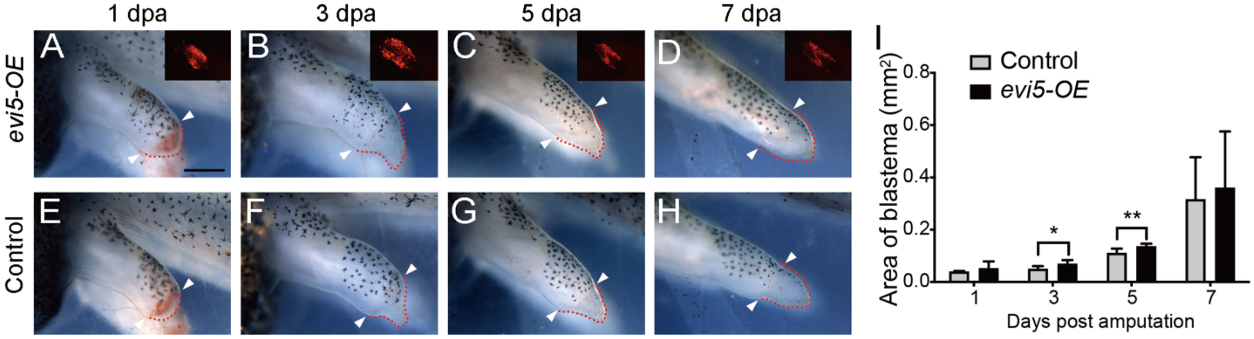

Figure S8. Overexpression of *evi5* in the hind limbs of NF stage 54-55 tadpoles. (A-H) Injection of the *evi5*-2A plasmid into the hind limbs of tadpoles at NF stage 54-55 showed that *evi5* overexpression promoted tissue growth but not enough to induce regeneration. **(I)** The area of the regenerated blastema was measured and analyzed for significance by t-test. Scale bar: 500 µm. * indicates $p < 0.05$ and ** indicates $p < 0.01$.

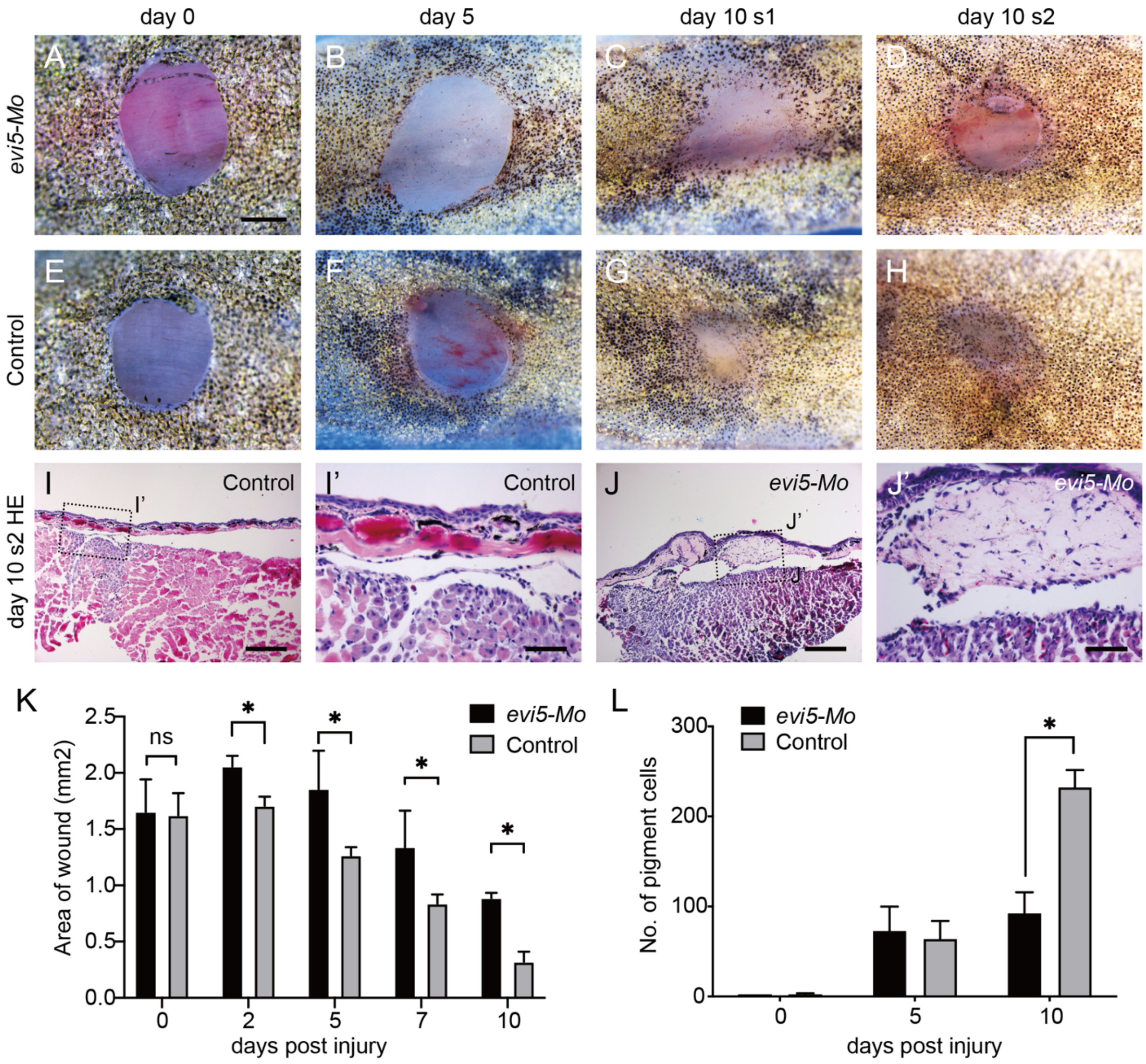

Figure S9. Effect of *evi5* Mo on wound healing in the *Xenopus* skin punch model. (A-H) Skin punches performed in the hindlimb of the *Xenopus* froglets. **(I, J)** HE staining of the control and Mo groups at day 10. **(I', J')** Enlargements of the squared areas in (**I**) and (**J**), respectively. **(K)** Measurement of the area of punch. **(L)** Counting of the number of pigment cells in wounds. Scale bars represent 500 μm in (**A-H**), 200 μm in (**I**) and (**J**), and 50 μm in (**I'**) and (**J'**). * indicates $p < 0.05$. One-way ANOVA, and multiple between-group t-test. Skin punches were performed with a 1 mm diameter biopsy punch (Acuderm Inc., USA).

Supplementary Methods

Xenopus limb cell culture and EdU incorporation assay

Xenopus tadpoles were euthanized with an overdose of MS222, and then immersed in 5% bleach for 30 seconds, followed by a thorough rinse with distilled water, to disinfect the skin. Tadpoles were then rinsed with PBS containing antibiotics (1× anti-anti, 100 µg/mL ampicillin, 100 µg/mL gentamicin, Sigma-Aldrich) and transferred to a new clean Petri dish, and the hindlimbs were removed with a clean razor blade and the skin peeled off with a pair of clean forceps. In a tissue culture hood, the blastema and limb stump connective tissues were cut into fine pieces after a brief rinse with culture medium (0.7 × DMEM) containing antibiotics. The tissue pieces were transferred onto a 60 mm tissue culture dish for attachment to a scratched surface, and cultured in 5 mL of *Xenopus* cell culture medium (0.7 × DMEM, 10% FBS), in a 28°C incubator (5% CO_2). Cells migrating out of the tissue blocks were collected by trypsinization with 0.25% trypsin/EDTA, filtered through a 40 µm cell strainer, pelleted, and then resuspended for passaging. Passages 2-3 of *Xenopus* limb cells were used for electroporation with Mo, followed by EdU incorporation assays.

For EdU incorporation analysis, 10 µM of EdU (5-ethynyl-2'-deoxyuridine, Thermo Fisher Scientific, A10044) was added into the cell culture 24 hours before detection of EdU incorporation by Click-it EdU imaging kit (Thermo Fisher Scientific, C10337) according to the manufacturer's instruction.

Alizarin red and Alcian blue skeletal staining

Tadpoles were euthanized with MS222 and eviscerated, washed briefly with water, and then fixed in 95% ethanol for 12-48 hours. Specimens were stained in 0.03% Alcian blue (Millipore Sigma, St. Louis, USA) until the cartilage was blue. After washing with 95% ethanol, specimens were cleared in a 1% KOH solution and then stained in 0.03% Alizarin solution (Millipore Sigma, St. Louis, USA). The specimens were washed in 1% KOH: 20% glycerol and 1:1 glycerol: 95% ethanol before being imaged.

Froglet hindlimb skin punches

Young adult *Xenopus* (>3 cm snout-vent) were anesthetized in 0.2% MS222. The dorsal skin of the hindlegs was punched with a 1 mm diameter biopsy punch (Acuderm Inc., USA). Punch depth was determined empirically to minimize the damage to the hindlimb muscles.
